# Itch suppression in mice and dogs by modulation of spinal α2 and α3GABA_A_ receptors

**DOI:** 10.1038/s41467-018-05709-0

**Published:** 2018-08-13

**Authors:** William T. Ralvenius, Elena Neumann, Martina Pagani, Mario A. Acuña, Hendrik Wildner, Dietmar Benke, Nina Fischer, Ana Rostaher, Simon Schwager, Michael Detmar, Katrin Frauenknecht, Adriano Aguzzi, Jed Lee Hubbs, Uwe Rudolph, Claude Favrot, Hanns Ulrich Zeilhofer

**Affiliations:** 1 Institute of Pharmacology and Toxicology, University of Zürich, Winterthurerstrasse 190, CH-8057 Zürich, Switzerland; 2 Neuroscience Center Zürich, Winterthurerstrasse 190, CH-8057 Zürich, Switzerland; 3 Drug Discovery Network Zürich (DDNZ), Winterthurerstrasse 190, CH-8057 Zürich, Switzerland; 4 Dermatology Department, Clinic for Small Animal Internal Medicine, Vetsuisse Faculty, Winterthurerstrasse 260, CH-8057 Zürich, Switzerland; 5 Institute of Pharmaceutical Sciences, Swiss Federal Institute of Technology (ETH) Zürich, Vladimir-Prelog-Weg 1-5/10, CH-8093 Zürich, Switzerland; 6 Institute of Neuropathology, University of Zürich and University Hospital Zürich, Schmelzbergstrasse 12, CH-8091 Zürich, Switzerland; 7 Laboratory of Organic Chemistry, Swiss Federal Institute of Technology (ETH) Zürich, Vladimir-Prelog-Weg 1-5/10, CH-8093 Zürich, Switzerland; 8 Laboratory of Genetic Neuropharmacology, McLean Hospital, 115 Mill Street, Belmont, MA 02478 USA; 9 Department of Psychiatry, Harvard Medical School, 401 Park Drive, Boston, MA 02215 USA

## Abstract

Chronic itch is a highly debilitating condition affecting about 10% of the general population. The relay of itch signals is under tight control by inhibitory circuits of the spinal dorsal horn, which may offer a hitherto unexploited therapeutic opportunity. Here, we found that specific pharmacological targeting of inhibitory α2 and α3GABA_A_ receptors reduces acute histaminergic and non-histaminergic itch in mice. Systemic treatment with an α2/α3GABA_A_ receptor selective modulator alleviates also chronic itch in a mouse model of atopic dermatitis and in dogs sensitized to house dust mites, without inducing sedation, motor dysfunction, or loss of antipruritic activity after prolonged treatment. Transsynaptic circuit tracing, immunofluorescence, and electrophysiological experiments identify spinal α2 and α3GABA_A_ receptors as likely molecular targets underlying the antipruritic effect. Our results indicate that drugs targeting α2 and α3GABA_A_ receptors are well-suited to alleviate itch, including non-histaminergic chronic itch for which currently no approved treatment exists.

## Abstract (web summary)

Chronic itch affects about 10% of the general population, however current treatments are largely ineffective. Here, the authors show that targeting of inhibitory α2 and α3GABA_A_ receptors reduces itch in mice and in a canine model, suggesting this a potentially useful therapeutic approach.

## Introduction

Chronic itch affects between 4–17% of the general population^1,2^. Most drugs currently used to treat itch are histamine H1 and H4 receptor blockers that work well against acute itch. By contrast, chronic itch is mostly histamine-independent and largely irresponsive to these medications^3^. Frequent causes of histamine-independent itch include, besides atopic dermatitis, cholestatic liver disease, end stage kidney failure, and opioid-therapy^4^. Drugs used to treat itch in these conditions include immune suppressants and drugs acting at the CNS such as gabapentinoids, antidepressants, and opioid receptor antagonists. In the majority of cases, these treatments do not provide adequate relief or cause severe side effects^5^.

Pruritic (itch) stimuli are detected by sensory neurons (primary pruritoceptors) that innervate the skin and transform these stimuli into electrical signals, i.e., action potentials. These action potentials are then relayed via the peripheral and central axons of primary pruritoceptors to central neurons in the spinal or medullary dorsal horn^6^. Only recently have researchers begun to understand the signaling molecules, receptors, transmitters and neuronal pathways of itch. Several “new” G protein-coupled receptors expressed by primary sensory neurons have been identified that are activated by pruritogens. One such receptor is the mas-related G protein coupled receptor A3 (MrgprA3 in mouse, or MRGPRX1 in human), which is activated by the antimalarial drug chloroquine^7^. Sensory neurons expressing this receptor become excited by a wide variety of pruritogens involved in acute histaminergic and non-histaminergic itch, as well as in chronic itch^8^. Other work addressed neuronal pathways involved in the spinal relay of itch. These studies identified excitatory interneurons expressing gastrin releasing peptide (GRP)^9^ or the GRP receptor (GRPR)^10,11^ as key elements of this process. These itch-relay pathways appear to be under tight control by dorsal horn inhibitory neurons. Lack of a certain subset of these neurons that depend on the transcription factor Bhlhb5 leads to severe chronic itch in mice^12^. Local ablation of inhibitory neurons of the deep dorsal horn induces abnormal grooming and biting behavior, and localized hair loss reminiscent of chronic itch syndromes^13^. Conversely, local activation of these neurons through DREADDs (designer receptors exclusively activated by designer drugs^14^) suppressed histamine-dependent and histamine-independent itch, demonstrating that inhibitory dorsal horn neurons exert a profound control over spinal itch relay^13^.

Inhibitory neurons of the spinal dorsal horn release two fast amino acid transmitters, GABA and glycine, to reduce the excitability of their postsynaptic target neurons. In the present study, we focused on the GABAergic system and investigated whether itch, in particular chronic itch, can be suppressed through pharmacological modulation of specific subtypes of spinal GABA_A_ receptors (GABA_A_Rs). GABA_A_Rs are pentameric anion channels built from a repertoire of 19 subunits^15^. Most GABA_A_Rs in the brain and spinal cord are composed of α, β, and γ subunits in a 2:2:1 stoichiometry. The mammalian genome harbors 12 genes encoding for these subunits (α1-6, β1-3, and γ1–3). Spinal GABA_A_Rs mainly contain α1, α2, α3, or α5 subunits together with β2/3 subunits and a γ subunit. α4 and α6 subunits are only sparsely expressed or completely lacking^16,17^. Differences in the physiological functions and pharmacological properties of these GABA_A_Rs are mainly determined by the α subunit^18^. In the present study, we first used genetically modified mice to identify α2/α3 containing GABA_A_Rs as key elements of spinal itch control. Building on this result we assessed potential antipruritic actions of an α2/α3GABA_A_R selective compound (TPA023B; ref. ^19^) and showed that it not only reduced acute histamine-dependent and histamine-independent itch in mice but also chronic itch in mice and dogs without apparent side effects.

## Results

### Inhibitory input to peripheral and spinal pruritoceptors

**Fig. 1:**
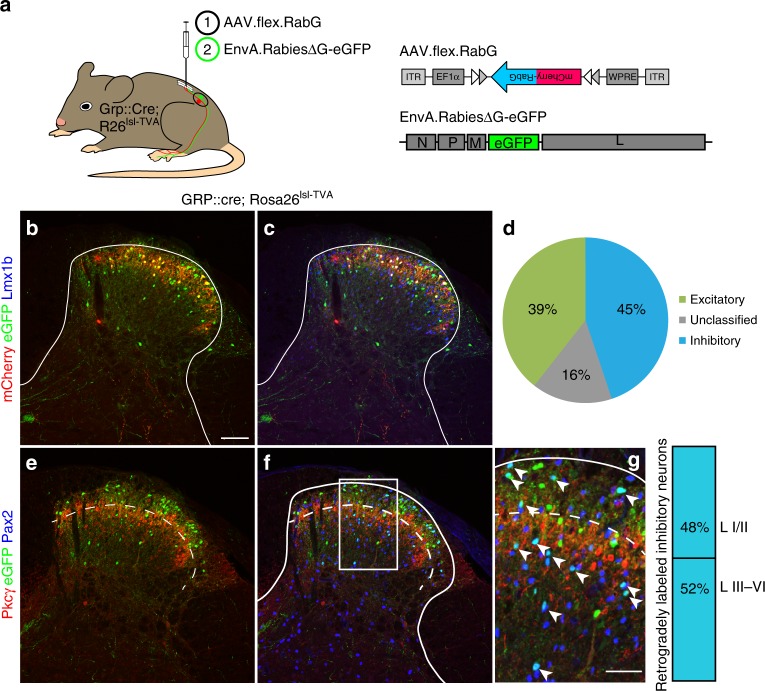
Inhibitory input onto spinal GRP positive itch relaying interneurons. **a** Neurons providing synaptic input onto GRP positive neurons were identified using transsynaptic rabies virus based tracing. GRP::cre;ROSA26^TVA^ mice (*n* = 3) were first injected with AAV.flex.mCherry-RabG into the left lumbar spinal cord, and 14 days later with an EnvA pseudotyped glycoprotein deficient rabies virus (EnvA.RabiesΔG-eGFP). **b**–**g** Transverse sections of the injected dorsal horn. Cre positive GRP neurons infected with AAV.flex.RabG (red) primary and secondary Rabies virus infected neurons (green). Co-staining with Lmx1b and Pax2 revealed excitatory and inhibitory neurons, respectively. **b** primary rabies virus infected neurons express eGFP and mCherry, and appear yellow. Secondary rabies virus infected neurons are green only. **c** Co-staining with Lmx1b (blue) reveals the excitatory population of secondary infected (transsynaptically labeled) neurons (magenta; eGFP and Lmx1b positive but mCherry negative) **d** Classification of transsynaptically labeled neurons as excitatory (39%, Lmx1b positive) or inhibitory (45%, Pax2 positive). 16% of transsynaptically infected neurons (eGFP positive but mCherry negative) were neither stained with antibodies against Lmx1b or Pax2, and remained unclassified. **e** Co-staining with PKCγ (red) indicates border between lamina II and III. mCherry was not visible in e-g because it required signal amplification by an mCherry antiserum. **f** Co-staining against Pax2 revealed transsynaptically labeled inhibitory neurons (magenta; Pax2 and eGFP positive). Scale bar, 100 µm. We did not find any mCherry/eGFP/Pax2 triple positive neurons (not shown) consistent with the known excitatory phenotype of spinal GRP neurons^22^. **g** Higher magnification of the area indicated in **f**. Arrow heads indicate rabies virus infected (eGFP positive) inhibitory (Pax-2 positive) neurons. Of the inhibitory transsynaptically labeled neurons, 48% were in laminae I/II and 52% in laminae III/IV. Quantifications were performed on 9 sections obtained from 3 mice

We first verified that itch relaying GRP neurons receive input from local inhibitory interneurons. Retrograde mono/transsynaptic rabies virus-based tracing experiments^20^ initiated from GRP::cre neurons identified numerous inhibitory and excitatory neurons presynaptic to GRP neurons (Fig. 1). About half of the inhibitory neurons were located in lamina II of the dorsal horn, where most inhibitory neurons are purely GABAergic^21^. The other half resided in deeper layers, where most inhibitory neurons co-express glycine and GABA^13^.

**Fig. 2:**
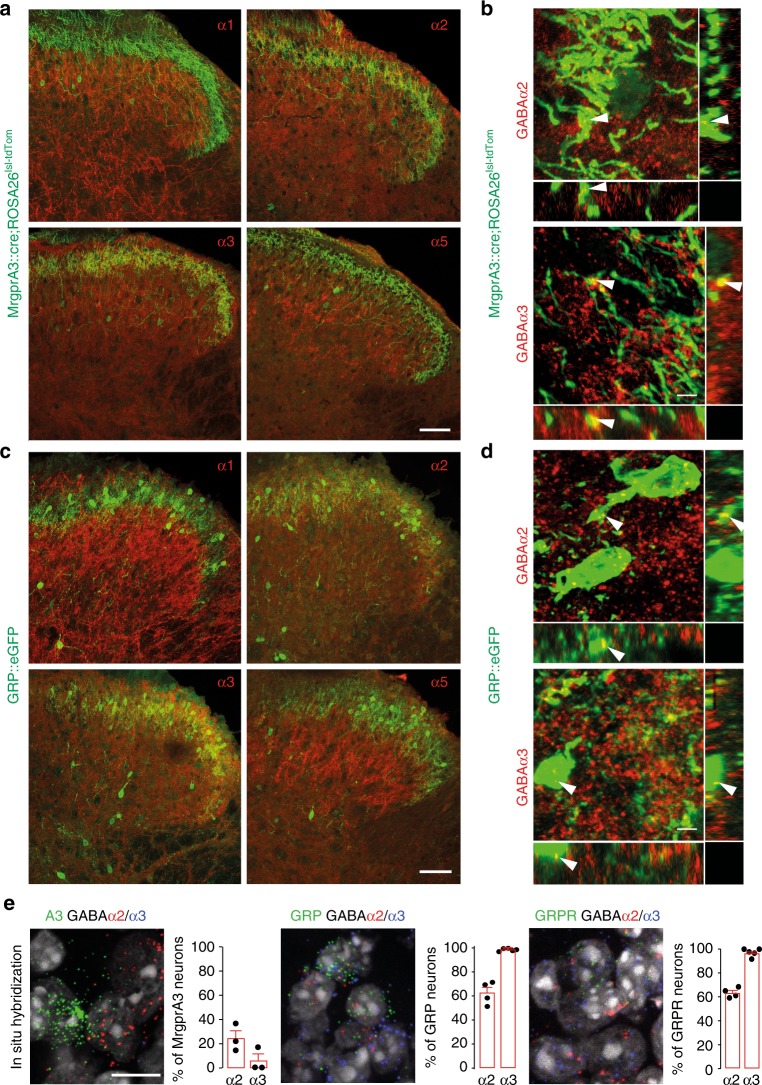
α2 and α3GABA_A_Rs are expressed on key elements of a spinal itch relay circuit. Expression of GABA_A_R α subunits in MrgprA3 positive primary pruritoceptors (**a**, **b**) and GRP positive dorsal horn neurons (**c**, **d**). **a**–**d** show transverse sections of the lumbar spinal cord of two MrgprA3::cre;ROSA26^lsl-tdTom^ and three GRP::eGFP transgenic mice stained with antibodies against α1, α2, α3, and α5GABA_A_R subunits. td-Tom and eGFP are shown in green, GABA_A_R α subunits in red. Overlapping expression (light green/yellow) of GABA_A_R α subunits with tdTom and eGFP was seen for α2 and α3GABA_A_R subunits, but not for α1 and α5GABA_A_R subunits. **b**, **d** Confocal analyses. Orthogonal views (stacks of 17–35 sections (1024 × 1024 pixels) at 0.4 µm intervals) verify co-localization of α2 and α3GABA_A_R subunits with MrgprA3 positive fibers and terminals (**b**) and GRP positive dorsal horn neurons (**e**) at higher magnification. Arrowheads indicate examples of co-localization. Scales bars, 50 µm (**a**, **c**), 5 µm (**b**, **d**). **e** Fluorescent in situ hybridization signals of α2 (red) and α3 (blue) subunits together with eGFP (to detect MrgprA3 neurons DRG neurons in MrgprA3::cre-eGFP transgenic mice), GRP and GRPR in situ hybridization signals (green, in wild-type mice). DAPI staining (gray) was used to indicate the location of cells. Bar charts: percent GABA_A_R α subunit positive neurons among the marker (MrgprA3, GRP, and GRPR) positive neurons. Each data point represents one mouse. Sections were obtained from 3–5 mice. Scale bar, 20 µm

We next investigated the presence of α1, α2, α3, and α5GABA_A_R subunits on spinal axon terminals of primary MrgprA3 positive pruritoceptors and on spinal GRP neurons (Fig. 2). Both MrgprA3 fibers and GRP neurons are concentrated in lamina II^8,22^, which also harbors α2 and/or α3GABA_A_R subunits at high density^16,17,23^. To visualize MrgprA3 axons and terminals and GRP neurons, we used GRP::eGFP and MrgprA3::cre-eGFP;ROSA26^lsl-tdTom^ transgenic mice. Immunostaining of spinal cord sections of these mice confirmed that the region of α2 and α3GABA_A_R subunit expression overlapped with that of MrgrpA3 terminals and GRP neurons (Fig. 2a,c). A similar GABA_A_R subunit expression pattern was found in the cervical spinal cord and the medullary dorsal horn (see Supplementary Fig. 1). By contrast, α1 and α5GABA_A_R subunits were largely missing from lamina II. Confocal analysis at higher magnification further demonstrated that α2 and α3GABA_A_R subunits were located on MrgprA3 fibers and GRP neurons (Fig. 2b,d). To allow a quantification of α2 and α3GABA_A_R subunit expressing neurons, we performed fluorescent in situ hybridization in sections of lumbar dorsal root ganglia (DRGs) and lumbar spinal cords (Fig. 2e). In these experiments, we also included spinal cord sections from GRPR::eGFP transgenic mice. Only about 20% of MrgprA3 positive DRG neurons expressed α2 and α3GABA_A_R subunit transcripts. This low expression is consistent with previously published single cell RNAseq data^24^ (Supplementary Table 1). By contrast, virtually all GRP and GRPR neurons expressed α3GABA_A_R subunit transcripts, and more than 60% of these neurons also expressed α2GABA_A_R subunits. Co-expression of α2 and α3GABA_A_R subunit transcripts with GRP and GRPR transcripts was also observed in human spinal cord tissue (Supplementary Fig. 2).

### GABA_A_R subtypes with antipruritic efficacy

**Fig. 3:**
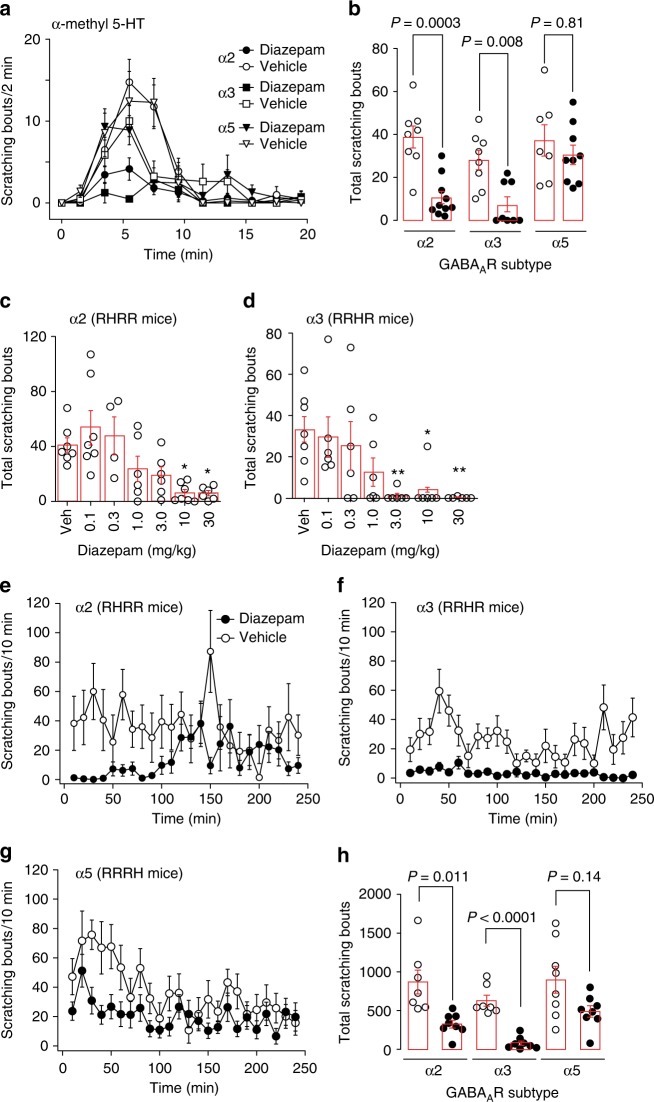
Antipruritic effects of GABA_A_R modulation in GABA_A_R triple point mutated mice. **a**–**d** Suppression of acute itch by α2, α3, and α5GABA_A_Rs activation (diazepam 10 mg kg^−1^, p.o., given 60 min prior to pruritogen injection) in three lines of triple GABA_A_R point mutated mice (α2 [RHRR mice], α3 [RRHR], or α5 [RRRH]). **a** Number of scratching bouts over time after diazepam injection. **b** Comparisons were made for diazepam (filled circles) versus vehicle (open circles) for the three genotypes. *P* values were obtained from unpaired two-sided t-tests corrected for three independent comparisons. *n* = 8 and 10 (α2, vehicle, diazepam); *n* = 8 and 8 (α3, vehicle, diazepam); *n* = 7 and 9 (α5, vehicle, diazepam). **c** Dose-dependence of the antipruritic effects of diazepam in RHRR mice (only α2GABA_A_Rs sensitive to diazepam). ANOVA followed by Dunnett’s post hoc test F(6,36) = 6.02; *P < 0.05; **P < 0.01, *n* = 7, 7, 4, 6, 6, 7, 5, for vehicle, and 0.1, 0.3, 1.0, 3.0, 10, and 30 mg kg^−1^. **d** Same as **c** but RRHR mice (only α3GABA_A_Rs sensitive to diazepam). ANOVA followed by Dunnett’s post hoc test F(6,37) = 4.42; *P < 0.05; **P < 0.01, *n* = 7 (vehicle) and 6 for all other groups. **e**–**h** Suppression of chronic itch. Antipruritic effects of α2, α3, and α5GABA_A_Rs in the oxazolone model of atopic-like dermatitis. Mice were sensitized to oxazolone over 17 days and treated with diazepam (10 mg kg^−1^, i.p.) or vehicle on day 18. Scratching bouts were counted for 6 h starting 15 min after drug or vehicle administration. **e**–**g** Number of scratching bouts plotted versus time after drug or vehicle administration. **h** *P* values obtained from unpaired two-sided *t*-tests for the three genotypes, corrected for three independent comparisons. Unpaired two-sided *t*-tests, *n* = 7 and 8 (α2, vehicle, diazepam); *n* = 7, 8 (α3, vehicle, diazepam); *n* = 8, 8 (α5, vehicle, diazepam). Error bars indicate s.e.m.

We then asked whether pharmacological targeting of GABA_A_Rs containing α2 or α3 subunits (α2/α3GABA_A_Rs) reduces itch. To this end, we administered diazepam, a classical benzodiazepine that non-selectively potentiates the activation of benzodiazepine-sensitive GABA_A_Rs. To restrict diazepam’s action to a single GABA_A_R subtype (α1, α2, α3, or α5), we used triple GABA_A_R point mutated mice, which carry a histidine to arginine (H → R) point mutation in three of the four benzodiazepine sensitive GABA_A_R α subunits^23^ (designated as HRRR, RHRR, RRHR, and RRRH mice, for mice in which only α1, α2, α3, or α5GABA_A_Rs remained diazepam sensitive). Because mice of this particular genetic background (129SvJ) have not yet been systematically analyzed in itch experiments, we first assessed their sensitivity to different pruritogens. We found that injection of α-methyl serotonin (α-methyl 5-HT), a metabolically more stable derivative of the pivotal itch messenger serotonin^25^, induced robust dose-dependent scratching behavior (Supplementary Fig. 3). We then tested the effect of systemic diazepam (10 mg kg^−1^, p.o.) on scratching responses evoked by α-methyl 5-HT (20 µg). Selective targeting of α2 or α3GABA_A_Rs (in RHRR or RRHR mice) strongly reduced scratching bouts (Fig. 3a–d). No significant reduction was observed after activation of α5GABA_A_Rs (in RRRH mice). A possible contribution of α1GABA_A_Rs could not be addressed as their activation induces strong confounding sedation^23,26^.

To test whether also chronic itch would respond to α2/α3GABA_A_R facilitation, we employed the oxazolone model of chronic atopic-like dermatitis^27^ (Fig. 3e–h). Oxazolone was repeatedly applied to the shaved nape of the neck for 17 days. This procedure induced profound scratching responses in wild-type mice and the three lines of triple GABA_A_R point mutated mice. Systemic treatment with diazepam (10 mg kg^−1^, i.p.) again strongly reduced scratching when only α2 or α3GABA_A_Rs were targeted (in RHRR and RRHR mice). It had no obvious effects on home cage behavior in these mice (see Supplementary Movies 1-4).

### Antipruritic efficacy of an α2/α3 selective GABA_A_R modulator

**Fig. 4:**
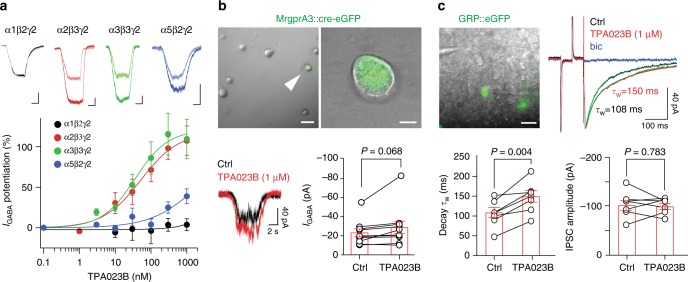
In vitro pharmacological profile of TPA023B. **a** Positive allosteric modulation by TPA023B of α1β2γ2, α2β3γ2, α3β3γ2, and α5β2γ2 GABA_A_Rs in HEK293 cells transiently transfected with the respective GABA_A_R subunits. Current traces (top) evoked by exogenous application of GABA at EC_5_. Light traces, control; bold traces, TPA023B (1 µM). Scale bars, 2 s, 200 pA. *n* = 6, for α2β3γ2, *n* = 5 for all other subtypes. **b** Potentiation of GABA_A_R currents in MrgprA3::cre-eGFP positive lumbar DRG neurons. Top: micrographs. Superposition of eGFP fluorescence and phase contrast image. Scale bars, 50 µm (left) and 10 µm (right). Bottom: examples of current traces under control conditions (black) and in the presence of TPA023B (1 µM) (red). Statistical analyses: GABAergic current amplitudes. Paired two-sided *t*-test; *n* = 10. **c** Potentiation of GABAergic inhibitory postsynaptic currents (IPSCs) in transverse spinal cord slices. Top left: Superposition of eGFP fluorescence and infrared gradient contrast in lamina II with two GRP::eGFP positive neurons, the recording patch pipette (right) and the stimulation electrode (left). Scale bar, 20 µm. Top right: Examples of GABAergic IPSCs (averages of 10 consecutive current traces) recorded under control conditions (ctrl, black), in the presence of TPA023B (red) and bicuculline (bic 10 µM, blue). Superimposed in green are fits to double exponential functions. (bottom) Statistical analyses. Changes in the weighted time constants (τ_w_) of IPSC decay and IPSC amplitudes. Bars and error bars (red) are mean ± s.e.m.. *P* values have been obtained from paired two-sided *t*-tests. *n* = 7 for both analyses. Error bars indicate s.e.m.

We then tested whether the data obtained in genetically modified mice would translate into therapeutic efficacy of GABA_A_R subtype-selective compounds. To this end, we tested the antipruritic efficacy of the α1-sparing GABA_A_R modulator TPA023B (ref. ^19^). As a prerequisite, we verified the in vitro pharmacological profile of TPA023B in transiently transfected HEK293 cells. As reported previously^19^, TPA023B had partial agonistic activity at the benzodiazepine binding site of α2β3γ2 and α3β3γ2 GABA_A_Rs, but did not potentiate α1β2γ2 GABA_A_Rs and had very weak potentiating effects on α5β2γ2 GABA_A_Rs (Fig. 4a). It did not activate GABA_A_Rs in the absence of GABA. We then asked whether this partial agonistic activity would translate to a facilitation of GABAergic inhibition in MrgprA3 or GRP neurons (Fig. 4b, c). In dissociated MrgprA3::cre-eGFP positive DRG neurons, TPA023B (1 µM) led to a slight statistically insignificant increase in GABA_A_R current amplitudes (*P* = 0.068, two-sided paired *t*-test, *n* = 10) (Fig. 4b). A more pronounced effect was observed in GRP dorsal horn neurons in which TPA023B (1 µM) significantly prolonged the decay of GABAergic inhibitory postsynaptic currents by 43 ± 10% (*P* < 0.01, paired two-sided *t*-test, *n* = 7) (Fig. 4c). Together with the in situ hybridization data, these results suggest that GABA_A_Rs inhibit the spinal relay of itch signals primarily at the level of dorsal horn interneurons rather than via inhibition of primary pruritoceptors.

**Fig. 5:**
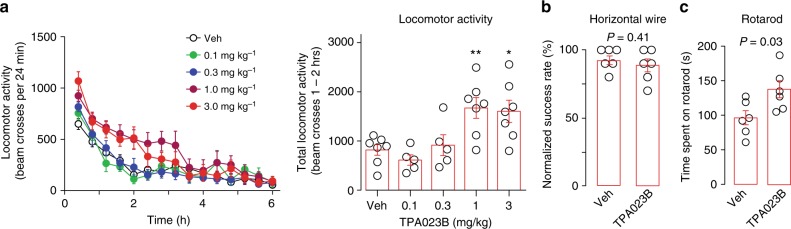
In vivo pharmacological profile of TPA023B. Behavioral effects of TPA023B in wild-type mice determined 1–2 h after p.o. drug administration. **a** Locomotor activity (number of beam crosses per time intervals of 24 min) was recorded for 6 h after TPA023B and plotted versus time after drug application. Statistical analysis of total number of beam crosses within the time interval 60–120 min. ANOVA followed by Dunnett’s post host test F(4,26) = 6.78. *P < 0.05; **P < 0.01, *n* = 7, 6, 5, 7, 7 for vehicle, and 0.1, 0.3, 1, and 3 mg kg^−1^. **b** Muscle relaxation assessed in the horizontal wire test between 60–120 min after drug administration (*P* = 0.41, unpaired two-sided *t*-test, *n* = 6 mice per group). **c** Motor coordination (rotarod performance) tested during 1–2 h after TPA023B (1 mg kg^−1^, p.o.). Data points are the average time spent on the rotarod of each mouse. TPA023B treated mice performed better than vehicle treated mice (*P* = 0.03, paired two-sided *t*-test, *n* = 6 mice per group). Error bars indicate s.e.m.

Would the favorable in vitro profile of TPA023B translate into reduced propensity to side effects? Consistent with the lack of agonistic activity at α1GABA_A_Rs, TPA023B did not induce sedation at doses up to 3 mg kg^−1^ (p.o.), but instead increased locomotor activity at 1 and 3 mg kg^−1^ (Fig. 5a). TPA023B did not cause muscle relaxation (Fig. 5b) and did not impair motor coordination (Fig. 5c).

**Fig. 6:**
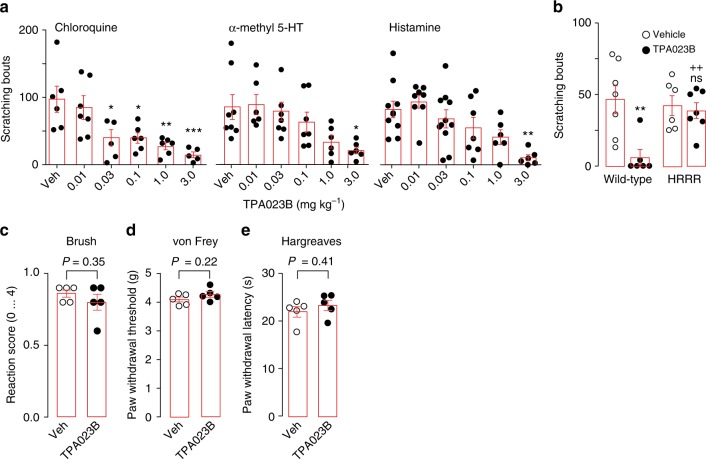
Antipruritic actions of TPA023B. **a** Antipruritic actions of TPA023B (p.o.) in mouse models of acute itch. Circles are total number of scratching bouts observed in individual mice within 30 min after intracutaneuos pruritogen injection. ANOVA, followed Dunnett’s post hoc test with vehicle as control. *P < 0.05, **P < 0.01, ***P < 0.001. Chloroquine (100 µg). F(5,29) = 6.44, *n* = 6, 7, 6, 6, 6, 5 for vehicle, and 0.01, 0.03, 0.1, 1.0, and 3.0 mg kg^−1^. α-Me5HT-evoked itch (20 µg). *n* = 8, 6, 7, 7, 6, 6 for vehicle, and 0.01, 0.03, 0.1, 1.0, and 3.0 mg kg^−1^. Histamine-evoked itch (100 µg). F(5,41) = 5.20, *n* = 9, 8, 11, 7, 6, 6 for vehicle, and 0.01, 0.03, 0.1, 1.0, and 3.0 mg kg^−1^. **b** The antipruritic action of TPA023B occurs via the benzodiazepine bind site of GABA_A_Rs. α-methyl 5-HT (20 µg) was injected intracutaneously into the right cheek. TPA023B (1 mg kg^−1^, p.o.) exerted strong antipruritic actions in wild-type mice. In HRRR mice, in which all TPA023B sensitive GABA_A_Rs subtypes had been rendered benzodiazepine-insensitive, TPA023B had completely lost its antipruritic action. Two-way ANOVA F(2,22) = 6.45. *P* = 0.019 for genotype × treatment. *P* = 0.005 (**) and 0.60 (ns) for treatment effect in wild-type and HRRR mice, respectively (*n* = 7, 6 (wild-type, vehicle and TPA023B); *n* = 6, 7 (HRRR mice, vehicle and TPA023B)). ^++^*P* < 0.01 relative to TPA023B-treated wild-type mice. **c**–**e** TPA023B (3 mg kg^−1^, p.o.) did not interfere with responses to somatosensory or acute noxious stimulation. **c** light mechanical stimulation with a paint brush, **d** punctate mechanical stimulation with von Frey filaments, **e** noxious heat stimulation with a radiant heat source. Five mice per group. Error bars indicate s.e.m.

We then continued investigating the efficacy of systemic (p.o.) TPA023B against acute itch evoked by α-methyl 5-HT (20 µg), chloroquine (100 µg), or histamine (100 µg) (Fig. 6a). Chloroquine-induced scratching was reduced by TPA023B in wild-type mice at doses ≥ 0.03 mg kg^−1^. Similar effects were obtained with α-methyl 5-HT and histamine. To verify that the antipruritic effect of TPA023B was due to its interaction with the benzodiazepine binding site of GABA_A_Rs, we used GABA_A_R triple point mutated mice (HRRR mice), in which all GABA_A_Rs susceptible to modulation by TPA023B (α2, α3, and α5 GABA_A_Rs) had been rendered benzodiazepine-insensitive. The antipruritic action of TPA023B was completely lost in these mice (Fig. 6b). We also confirmed that the H → R point mutation prevented potentiation and binding of GABA_A_Rs by TPA023B (Supplementary Fig. 4). TPA023B (3 mg kg^−1^, p.o.) had no significant effect on responses evoked by stimulation with a paint brush or von Frey filaments, and did not reduce responses to acute noxious heat (Fig. 6c–e) excluding a non-specific block of sensory relay through the spinal cord.

**Fig. 7:**
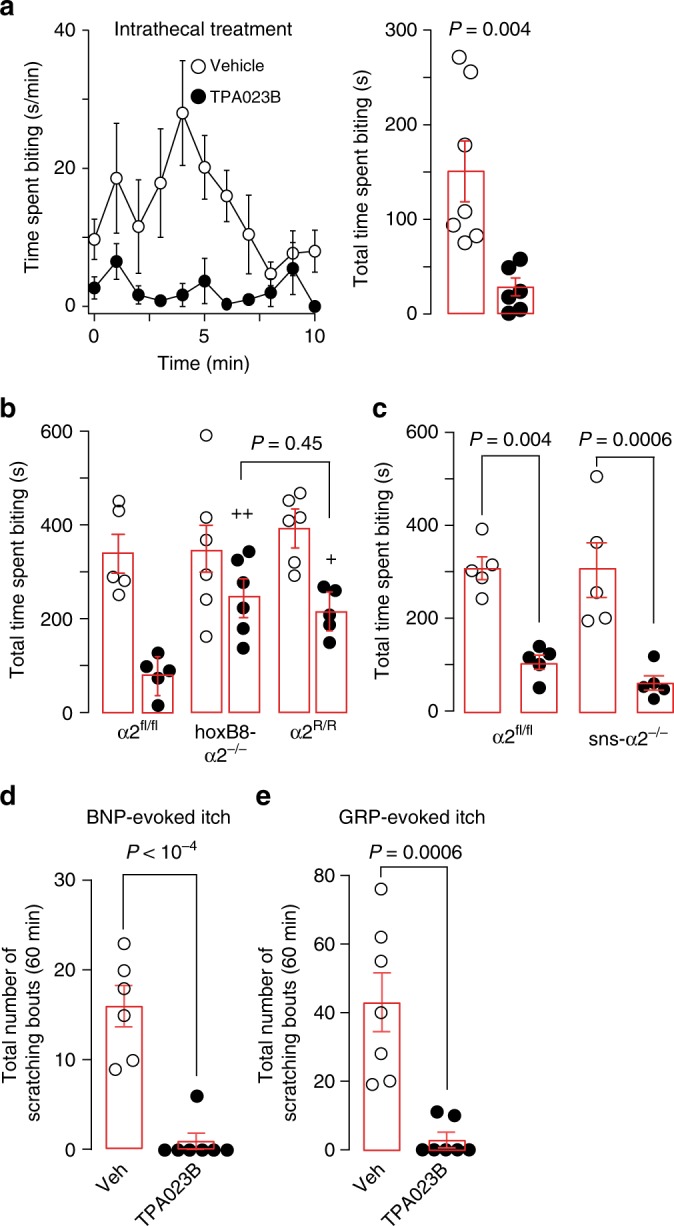
TPA023B reduces acute itch mainly through spinal α2GABA_A_Rs. **a** Antipruritic action of intrathecally injected TPA023B (0.3 mg kg^−1^) in wild-type mice. Chloroquine (100 µg) was injected intracutaneously into the left thigh. Time spent biting of the injected skin area (s min^−1^) and total number of scratching bouts counted during 0–10 min after pruritogen injection. *P* = 0.004, unpaired two-sided *t*-test, *n* = 7 and 6, for vehicle and TPA023B. **b** Antipruritic action of TPA023B (1 mg kg^−1^, p.o.) in hoxB8-α2^−/−^ mice (*n* = 6 and 6, for vehicle and TPA023B), GABA_A_R α2^fl/fl^ mice (*n* = 5 for both groups) and global α2GABA_A_R (H → R) point mutated mice (*n* = 6 and 5, for vehicle and TPA023B, respectively). Chloroquine (100 µg) was injected intracutaneously into the left thigh. Chloroquine-induced biting responses (total time spent biting the injected skin area) were virtually identical in all three genotypes (ANOVA followed by Bonferroni post-hoc test F(14,2) = 0.39, *P* = 1.0 for all comparisons). By contrast, biting responses in TPA023B (1 mg kg^−1^, p.o.) treated mice differed significantly between genotypes (ANOVA followed by Bonferroni post-hoc test F(2,13) = 10.6). ^+^*P* ≤ 0.05; ^+ +^*P* ≤ 0.01. No significant difference (*P* = 0.45) was found between hoxB8-α2^−/−^ mice and global α2 ^R/R^ mice indicating that at least the α2GABA_A_R-mediated component occurred through a spinal site. **c** Antipruritic actions of TPA023B (1 mg kg^−1^ p.o.) in sns-α2^−/−^ mice lacking α2GABA_A_Rs specifically from small diameter nociceptive and pruritoceptive DRG neurons, *n* = 5 for all groups. Chloroquine (100 µg) was injected intracutaneously into the left thigh. TPA023B exerted similar antipruritic actions in α2^fl/fl^ and sns-α2^−/−^ mice (ANOVA followed by Bonferroni post-hoc test F(1,16) = 0.36) indicating that peripheral α2GABA_A_Rs do not contribute to the antipruritic actions of TPA023B. **d**, **e** Antipruritic effects of TPA023B (3 mg kg^−1^, p.o.) against itch evoked by intrathecally injected BNP (10 nmoles) (*n* = 7 per group) (**d**) or GRP (1 nmole) (*n* = 6 and 7 mice, for TPA023B and vehicle) (**e**). Unpaired two-sided t-tests. Error bars indicate s.e.m.

Subsequent experiments with intrathecal injection of TPA023B (0.3 mg kg^−1^) at the level of the lumbar spinal cord in wild-type mice (Fig. 7a) confirmed that the antipruritic action of TPA023B originated from the spinal cord. Further support was obtained with hoxB8-GABA_A_Rα2^−/−^ (Fig. 7b) and sns-GABA_A_Rα2^−/−^ mice (Fig. 7c). These conditional knock-out mice lack α2GABA_A_Rs either from all spinal cord and DRG neurons (hoxB8-GABA_A_Rα2^−/−^)^28^ or only from small diameter Nav1.8 positive (nociceptive and pruritoceptive) DRG neurons^29^. Antipruritic effects of systemic TPA023B (3 mg kg^−1^, p.o.) were strongly reduced in hoxB8-GABA_A_Rα2^−/−^ mice but remained unaltered in sns-GABA_A_Rα2^−/−^ mice. In line with an action on intrinsic dorsal horn neurons, TPA023B (3 mg kg^−1^, p.o.) also reduced scratching behavior elicited by intrathecal injection of brain-type natriuretic peptide (BNP; 10 nmoles) and GRP (1 nmole) (Fig. 7d, e) consistent with an itch inhibitory effect occurring primarily via intrinsic dorsal horn neurons.

**Fig. 8:**
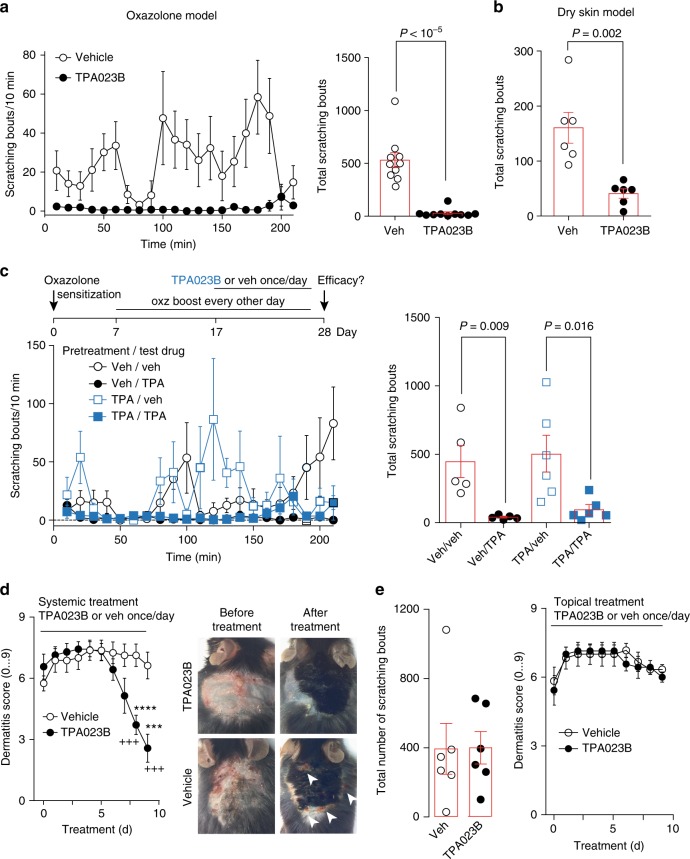
Antipruritic actions of TPA023B in chronic itch models. **a** Oxazolone model of chronic atopic-like dermatitis. Scratching bouts after injection of TPA023B (1 mg kg^−1^, i.p.) over time (left). Total numbers of scratching bouts between 15 and 210 min after TPA023B/vehicle administration (right). Unpaired two-sided *t*-test, *n* = 10 mice per group. **b** Same as **a** but dry skin model of dermatitis, *n* = 6 mice per group. **c** Lack of tolerance development after 10 day treatment with TPA023B (TPA, 1 mg kg^−1^, i.p. once daily). Two-way ANOVA (pretreatment x treatment), F(1,1) = 0.96, *P* = 0.34. Two-sided *t*-test indicates similar antipruritic effects in vehicle and TPA023B pretreated mice, *n* = 6 (TPA/TPA), *n* = 5 for all other groups. **d** Dermatitis score. Chronic systemic treatment with TPA023B (1 mg kg^−1^ i.p., once per day) starting on day 11 of oxazolone exposure. Oxazolone challenges were continued during TPA023B treatment every other day. Only mice with a dermatitis score of 5 or higher on day 11 were included. In TPA023B-treated mice the dermatitis score decreased from day 6 of treatment onwards (F(9,54) > 38.7; *P* < 0.026 to *P* < 0.001) (left), but remained almost constant in vehicle-treated mice (right). Mixed repeated measures ANOVA revealed a significant treatment x time interaction F(9,117) = 22.6; *P* < 0.001 for day 8 and 9 (^+++^). Differences between both treatment groups were significant at day 8 and 9. ***P = 0.0009 and ****P < 00001. Photographs show the same mice before treatment (left) and after 9 days of treatment (right). **e** Chronic topical treatment (0.3 µM TPA023B in 100 µl, once per day). Left: total number of scratching bouts between 15 and 210 min after TPA023B on day 9 after treatment begin. Unpaired two-sided t-test, *P* = 0.97, *n* = 6 mice per group. Right: same as left but dermatitis score (mixed repeated measures ANOVA treatment × time interaction F(9,99) = 0.61; *P* = 0.78; *n* = 6 and 7, for vehicle and TPA023B, respectively). Error bars indicate s.e.m.

Would TPA023B also alleviate chronic itch? We first employed again the oxazolone model of atopic-like dermatitis. Acute treatment with TPA023B (1 mg kg^−1^, i.p.) caused a highly significant reduction in the number of scratching bouts (determined during the interval 15–210 min after drug injection) (Fig. 8a; for potential effects of i.p. TPA023B on locomotor behavior and motor performance see Supplementary Fig. 5). A similar antipruritic action was observed in the dry skin dermatitis model^30^ (Fig. 8b). Treatment of chronic itch conditions with TPA023B would require that no loss of therapeutic activity occurs during repeated applications. To test whether TPA023B would retain therapeutic activity during chronic treatment, we compared the antipruritic activity of TPA023B in drug naïve mice with that in mice treated with TPA023B (1 mg kg^−1^ i.p.) once daily for ten days (Fig. 8c). No significant loss of antipruritic activity was observed. Treatment with TPA023B over several days also alleviated skin lesions. After seven days of once daily treatment with TPA023B (1 mg kg^−1^, i.p.), mice showed a progressive reduction in their dermatitis scores (Fig. 8d). Furthermore, chronic treatment with TPA023B also reduced the infiltration of the affected skin by macrophages (Supplementary Fig. 6). By contrast, topic treatment with TPA023B (100 µl 0.3 µM, once daily) over the same time period neither changed scratching behavior nor dermatitis scores (Fig. 8e).

**Fig. 9:**
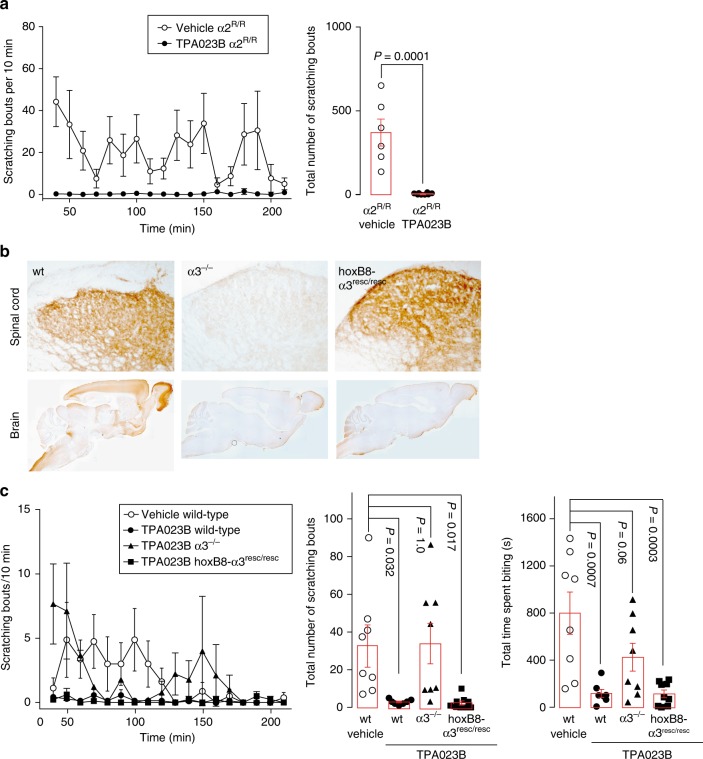
Spinal α3GABA_A_Rs are sufficient for full antipruritic efficacy of TPA023B in chronic itch. **a** Global H → R point mutation of α2GABA_A_Rs did not reduce the antipruritic effects of TPA023B (1 mg kg^−1^ i.p.) in the oxazolone model of atopic-like dermatitis. Unpaired *t*-test, *P* = 0.0001, *n* = 6 and 8, for vehicle and TPA023B, respectively. **b** HoxB8-cre-mediated rescue of spinal α3GABA_A_R expression in α3GABA_A_R^−/−^ mice. Immunoperoxidase staining of α3GABA_A_R subunits in transverse sections of the lumbar spinal dorsal horn (top) and in sagittal brain sections (bottom). **c** The antipruritic action of TPA023B was fully or partially (for scratching or biting behavior, respectively) lost in global α3GABA_A_R^−/−^ mice but completely recovered in hoxB8-α3^resc/resc^ mice (*P* = 1.0 wt/TPA023B versus hoxB8-α3^resc/resc^/TPA023B). Note that in these experiments, dermatitis was induced at the level of the mid lumbar back to ensure that skin territories were affected that are innervated by spinal cord segments with HoxB8-cre mediated rescue. ANOVA followed by Dunnett’s post hoc test F(3,28) = 5.80, F(3,28) = 8.72, for scratching bouts and time spent biting, respectively. *n* = 8, 7, 8, 9, for wt/veh, wt/TPA023B, α3^−/−^/TPA023B, and hoxB8-α3^resc/resc^/TPA023B, respectively. Error bars indicate s.e.m.

Additional experiments verified a spinal site of action also for chronic itch. First experiments in global α2 ^R/R^ point mutated mice revealed that eliminating diazepam sensitivity from α2GABA_A_Rs was not sufficient to block the antipruritic action of TPA023B (Fig. 9a). Because selective targeting of α3GABA_A_Rs had an even stronger antipruritic effect than targeting α2GABA_A_Rs (compare Fig. 3h), we focused our efforts on the α3GABA_A_R subtype and investigated α3GABA_A_R subunit knock-out mice, in which deletion of the α3GABA_A_R subunit gene was achieved through the insertion of a duplicated exon (5*) flanked by loxP sites^31^. This design allows a cre-dependent (tissue-specific) rescue of α3GABA_A_R subunit expression (Fig. 9b). The antipruritic effect of TPA023B was completely lost after global deletion of α3GABA_A_R subunits but was fully restored in spinal cord-specific hoxB8-GABA_A_Rα3^resc/resc^ mice (Fig. 9c).

### Antipruritic efficacy of TPA023B in pruritic dogs

**Fig. 10:**
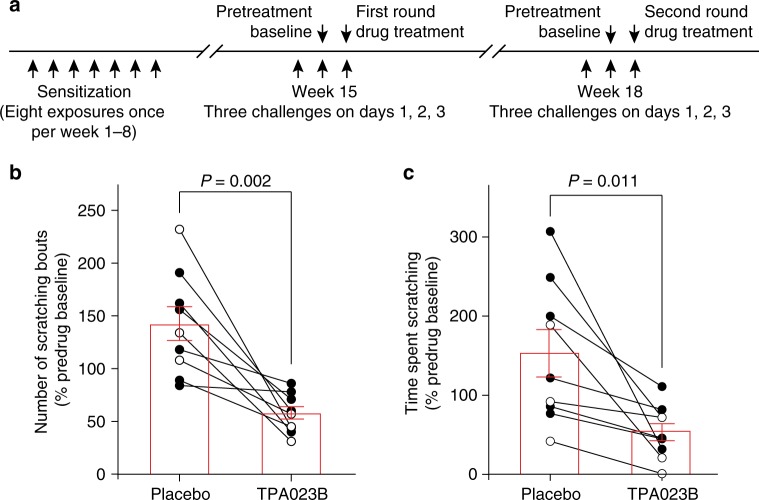
Antipruritic actions of TPA023B in a dog model of allergic itch. **a** Time line of the study. TPA023B was administered as a tablet (20 mg, p.o.) and dogs (*n* = 9) were monitored for 6 h starting immediately after drug/placebo administration. An additional round of three challenges (not displayed) was made on week 12. **b**, **c** Effect of TPA023B on the number of scratching bouts per hour (**b**) and time spent scratching in min hour^−1^ (**c**). Both outcome parameters were normalized to the values obtained on the day before drug treatment. Dots and connecting lines represent values of individual dogs and changes in these values following treatment with TPA023B. Open and closed circles are dogs that received TPA023B either in round 1 or in round 2, respectively. *P* values were determined using paired two-sided *t*-tests. Error bars indicate s.e.m.

Encouraged by its efficacy in mice and the absence of apparent side effects, we tested the antipruritic efficacy of TPA023B in a second, hierarchically higher, species (Fig. 10). We chose dogs because models of atopic dermatitis that closely mimic natural disease are well-established in this species^32^ and because the tolerability of TPA023B in dogs had already been established^33^. We performed a pseudo-randomized observer-blind placebo-controlled cross-over trial and in twelve beagle dogs sensitized to house dust mites by repeated exposure of their lower abdomen to lyophilized extracts of *Dermatophagoides farinae*^34^. After a sensitization period of 8 weeks during which the dogs were exposed to the extracts once a week, three challenges on three consecutive days were made in week 12. Nine of the 12 dogs developed clinical signs of pruritus and were included in subsequent experiments. These nine dogs were again challenged in weeks 15 and 18 on three consecutive days. The challenge on day 2 was used to obtain baseline values. The challenge on day 3 was followed by TPA023B (20 mg in one tablet, equivalent to about 2 mg kg^−1^, p.o.) or placebo administration. The same procedure was repeated in week 18 with a cross-over design. Both the time spent scratching and the numbers of scratching bouts were counted over 6 h after drug administration and normalized to the values on the day before drug exposure. Compared to placebo, a significant reduction was observed for both read-outs. Five of the 9 dogs (56%) responded with a reduction by more than 50% (for responses of the individual dogs see Supplementary Table 2). These results indicate that specific targeting of α2/α3GABA_A_Rs alleviates itch not only in mice but also in dogs supporting the potential for translation to more complex species, including possibly, also human patients.

## Discussion

Local application of TPA023B to the spinal cord and specific genetic ablation of spinal α2GABA_A_Rs have shown that most if not all of its antipruritic action originates from the spinal (or medullary) dorsal horn. This is consistent with our finding that virtually all GRP and GRPR neurons in the mouse spinal cord express α3GABA_A_R transcripts and more than 60% of these neurons also express α2GABA_A_Rs. Using post mortem tissue samples, we confirmed a similar coexpression of α2 and α3GABA_A_R subunits on GRP and GRPR neurons for the human spinal cord, in line with the highly conserved expression pattern of spinal GABA_A_R subunits in rodents and humans^35,36^.

MrgprA3 is primarily activated by chloroquine^7^. However, the fibers carrying this receptor respond to many pruritogens and convey signals related to both histaminergic and non-histaminergic itch^8^. A critical role of GRP^37^ and GRPR^38^ neurons in spinal itch circuits is meanwhile also well established. After local spinal ablation of GRP neurons, mice respond less to pruritic stimuli^37^. This is also supported by a study that employed BNP-conjugated saporin to ablate spinal GRP neurons^9^. Besides GRP and GRPR positive interneurons, neurokinin 1 (NK1) receptor expressing neurons of the dorsal horn are also relevant to spinal itch relay^39^. Previous work from our group has shown that these neurons also express α2 and α3GABA_A_Rs^40^.

α2/α3GABA_A_Rs do not only control itch but also pain^23,40^ (for a review see ref.^41^). The latter function is supported by the expression of α2/α3GABA_A_Rs receptors on sensory fibers and dorsal horn nociceptive neurons^16,40^ and also by the efficacy of spinal GABAergic neuron transplants against pain and itch^42,43^. Unlike opioids that reduce pain but cause itch^44^, drugs targeting α2/α3GABA_A_Rs should alleviate both itch and pain. Activation of α3GABA_A_R had a stronger impact on scratching responses than α2GABA_A_Rs. This ratio is reverse in case of analgesia^23,40^ in line with the view that sensory fibers and dorsal horn neurons processing itch or pain are not identical^3,45^. There were also differences in the contribution of α2 and α3GABA_A_Rs between models of acute and chronic itch. These may hint at potential neuroplastic changes occurring during the transition from acute to chronic itch.

In the present study, we have assessed unwanted effects that typically occur with classical non-selective benzodiazepine site ligands. TPA023B was devoid of sedative effects, did not impair motor coordination and did not lose antipruritic activity during prolonged treatment. The absence of a sedative effect is consistent with the pivotal role of α1GABA_A_Rs in benzodiazepine-induced sedation^26^. The increase in locomotor activity observed in the present study is possibly related to the anxiolytic action of α2GABA_A_Rs (see also ref.^23^). Previous studies in triple GABA_A_R point mutated mice have attributed the impairment of motor coordination and tolerance development to α1 or α3GABA_A_Rs^23^. The lack of these side effects in TPA023B treated mice may either come from the absence of activity at α1GABA_A_Rs or from the only partial agonistic activity (relative to the full agonist diazepam) at α3GABA_A_Rs.

In human patients, the most challenging type of pruritus is chronic non-histaminergic pruritus which is often due to liver or kidney failure or treatment with opioids^46^. While antihistaminergics provide good itch relief in cases associated with urticaria, other forms respond less well to these drugs or not at all. At present, these conditions are mostly treated off-label with systemically applied anticonvulsants, such as gabapentin and pregabalin, antidepressants, opioid antagonists, or more recently with NK1 receptor blockers (aprepitant), or immunosuppressants^47^. None of these compounds have been approved for systemic treatment of chronic itch conditions. In our study, TPA023B was similarly effective against non-histaminergic and histamine-induced itch, and, in general, more effective in chronic than in acute itch. If the antipruritic efficacy combined with good tolerability observed in the present study translates to human patients, TPA023B or related compounds should be well-suited for the treatment of chronic itch in humans.

In a broader context, our study adds to a growing body of evidence indicating that subtype selective GABA_A_R modulators promise not only better tolerability but may also open new avenues to the treatment of disorders that have hitherto not been considered indications for benzodiazepines. Such potential new opportunities include, in addition to itch and pain, cognitive enhancement by inverse agonists at α5 GABA_A_Rs^48,49^, depression and autism spectrum disorders (for a review see ref. ^50^). Given the wide-spread, almost ubiquitous, expression of GABA_A_Rs in the CNS, such new opportunities should not come as a surprise.

## Methods

### Mice

Homozygous triple and quadruple (H → R) GABA_A_R α subunit point-mutated mice were generated by cross breeding of single point-mutated mice^26,51,52^. GABA_A_R point mutated mice and the corresponding control mice were of the (129 × 1/SvJ) background. Other transgenic mice (including single GABA_A_R point mutated mice) and the corresponding control mice were of the C57BL/6 genetic background. BAC transgenic GRP::eGFP (Tg(Grp-EGFP)DV197Gsat/Mmucd), GRP::cre (Tg(Grp-cre)KH288Gsat/Mmucd) and GRPR::eGFP (Tg(Grpr-EGFP)PZ62Gsat/Mmucd) were obtained from the GENSAT project (http://www.gensat.org). BAC transgenic MrgprA3::cre-eGFP (Tg(Mrgpra3-GFP/cre)#Xzd)^8^ were generously provided by Dr. Xinzhong Dong, Johns Hopkins University. BAC transgenic mouse lines were maintained in the heterozygous state. TVA reporter mice (Gt(ROSA)26Sor < tm1(Tva)Dsa)^53^ expressing the TVA transgene from a ubiquitous promoter in a cre dependent manner were obtained from Dr. Dieter Saur (Technical University of Munich). (ROSA)26Sor^tm14(CAG-tdTomato)Hze^/J (ROSA26^lsl-tdTom^, also known as Ai14; www.jax.org/strain/007914) were obtained from Jackson Laboratories. Sns-cre mice (Tg(Scn10a-cre)1Rkun)^54^ were kindly provided by Dr. Rohini Kuner from University of Heidelberg. HoxB8-cre (Tg(Hoxb8-cre)1403Uze)^55^ and GABA_A_Rα3^−/−^ mice (Gabra3^tm2Uru^)^56^ have been described previously. Mice were genotyped with PCR (for primers see Supplementary Table 3). All animal experiments were complied with the relevant ethical regulations. Permission for all animal experiments was obtained from the Veterinäramt des Kantons Zürich (licenses 126/2012, 257/2014, ZH031/16, ZH113/16).

### Human spinal cord tissue

came from autopsies done at the Department of Neuropathology, University of Zurich. Both, tissue samples and data were anonymized. The analysis of the small tissue samples used in this study did not require special permission (confirmed by the Ethics Committee of the Canton of Zurich, BASEC Req-2017-01005).

### Drugs

Diazepam was obtained from Sigma. TPA023B (6,2′-difluoro-5′-[3-(1-hydroxy-1-methylethyl)imidazo[1,2-b][1,2,4]triazin-7-yl]biphenyl-2-carbonitrile) was synthesized by ANAWA, purity was >95%. For oral (p.o.) and intraperitoneal (i.p.) administration to mice, diazepam and TPA023B were suspended in 0.9% saline/1% Tween80. For electrophysiological experiments and radioligand binding, TPA023B was dissolved in DMSO and diluted with extracellular solution to 0.001–1 µM (final DMSO concentration ≤0.12%). For experiments in dogs, TPA023B was packed into tablets containing 20 mg TPA023B, 38 mg Prosolv^®^ SMCC90 (JRS Pharma) and 2 mg Ac-Di-Sol^®^ (FMC). Placebo tablets were of the same weight, size, color and composition but contained 2.4 mg Quinoline Yellow (E104) instead of 20 mg TPA023B. Quinoline Yellow was added to ensure the same color of TPA023B and placebo tablets. GRP and BNP were obtained from Tocris. For intrathecal (i.t.) administration into mice, both peptides were dissolved in distilled water and diluted in extracellular solution to 1–10 nmoles per 10 µl. For topical application, TPA023B (0.3 µM 100 µl) was suspended in acetone/olive oil (4:1 v/v) and applied on the shaved nape of the neck once daily.

### AAV preparation

AAV.flex.mCherry-2A-RabG vector was cloned in-house and packaged at Penn Vector Core (Perelman School of Medicine, University of Pennsylvania) using their custom service. AAV.flex.mCherry-2A-RabG vector was cloned by excising the ChR2-mCherry fusion protein from pAAV-Ef1a-DIO-hChR2(H134R)-mCherryWPRE-pA (kindly provided by Dr. Karl Deisseroth, Stanford University) with AscI and NheI and replacing it with PCR amplified mCherry-2A-RabG cDNA. AAV of serotype 1 vector was used in this study.

### Intraspinal virus injections

Animals were anesthetized with 2–5% isofluorane and lumbar vertebrae L4 and L5 were exposed. The animal was then placed in a motorized stereotaxic frame and the vertebral column was immobilized using a pair of spinal adaptors. The vertebral lamina and dorsal spinous process were removed to expose the L4 lumbar segment. The dura was perforated about 500 μm left of the dorsal blood vessel using a beveled 30 G needle. Viral vectors were injected at a depth of 200–300 μm using a glass micropipette (tip diameter 30–40 μm) attached to a 10 μl Hamilton syringe. The rate of injection (30 nl min^−1^) was controlled using a PHD Ultra syringe pump with a nanomite attachment (Harvard Apparatus, Holliston, MA). The micropipette was left in place for 5 min after the injection. Wounds were sutured and the animals were injected i.p. with 0.03 mg kg^−1^ buprenorphine and allowed to recover on a heat mat. Rabies virus injected mice were subjected to perfusion 3–5 days after injection.

### Retrograde tracing experiments

Retrograde monosynaptic tracing experiments were initiated from GRP::cre expressing neurons of the lumbar spinal cord. A two-step strategy was used. This involved first an injection of an AAV helper virus (AAV.flex.mCherry-2A-RabG; 2.9 × 10^9^ GC per injection in 300 nl) containing a bicistronic Cre-dependent mCherry and rabies glycoprotein (RbG) expression cassette, and fourteen days later a subsequent injection of an EnvA (avian sarcoma leukosis virus “A” envelop glycoprotein) pseudotyped glycoprotein-deficient rabies virus (EnvA.RabiesΔG.eGFP; 1 × 10^6^ GC per injection in 500 nl). The TVA protein expressed from the Rosa26 reporter mouse line^53^ enabled cell type specific infection of Grp::Cre positive neurons, and the RbG was expressed to transcomplement the glycoprotein-deficient rabies virus in primary infected neurons. For subsequent neurochemical analyses, mice were perfused with 4% paraformaldehyde (PFA) in PBS followed by postfixation in 4% PFA in PBS for 1–2 h five days after rabies virus injection. The tissue was cut into 25 μm thick coronal cryosections, which were mounted onto Superfrost Plus microscope slides (Thermo Scientific, Zurich, Switzerland). The following antibodies were used: rat anti-mCherry (1:1000; Molecular Probes; RRID:AB_2536611), rabbit anti-GFP (1:1000; Molecular Probes; RRIC:AB_221570), chicken anti-GFP (1:1000; Thermo Fisher Scientific, Waltham, MA, USA; RRID:AB_2534023), guinea pig anti-Lmx1b (1:10,000; gift from Carmen Birchmeier^57^) rabbit anti Pax2 (1:400; Invitrogen, Carlsbad, CA; USA; RRID:AB_2533990), anti PKCγ (1:1000; Santa Cruz Biotechnology, Dallas, TX, USA; RRID:AB_632234) and cyanine 3 (Cy3)-conjugated, Alexa Fluor 488-conjugated, DyLight 488-conjugated, 647-conjugated, and 649-conjugated donkey secondary antibodies (1:500; Dianova, Hamburg, Germany). Retrograde tracing experiments were done in 8–9-week-old mice.

### Image analysis

Fluorescent images were acquired on a Zeiss LSM710 Pascal confocal microscope using a 0.8 NA 20x Plan-apochromat objective or a 1.4 NA 63x EC Plan-Neofluar oil-immersion objective and the ZEN2012 software (Carl Zeiss). Whenever applicable, contrast, illumination, and false colors were adjusted in ImageJ or Adobe Photoshop (Adobe Systems, Dublin, Ireland). Cell numbers were quantified in a total of 9 sections prepared from 3 animals. In order to avoid double counting of cells in adjacent sections, all sections used for quantification were taken at a distance of at least 50 µm. The numbers of immune reactive cells were determined using the ImageJ Cell Counter plug-in.

### Immunohistochemistry and image analysis of GABA_A_R subunits

Colocalization of GABA_A_R α subunits with MrgprA3 axons and GRP neurons was visualized on 40 µm thick transverse mouse lumbar spinal cord cryosections. Mice were deeply anaesthetized with pentobarbital (nembutal, 50 mg kg^−1^, i.p.) and perfused with oxygenated aCSF. Spinal cords were rapidly collected by pressure ejection and placed in ice-cold 4% PFA for 90 min. The spinal cords were then cryoprotected overnight in a 30% sucrose/PBS solution, snap frozen with dry ice and cut in 40 μm thick coronal free-floating slices kept in antifreeze at −20 °C until the day of staining^16^. Antibodies were home-made subunit-specific antisera^16,58^. Final dilutions were 1:20,000 (α1), 1:1,000 (α2), 1:10,000 (α3), and 1:3,000 (α5). The distribution of GABA_A_R α subunits in dorsal horn GRP neurons and MrgprA3 axons was analyzed by immunofluorescence staining on coronal sections prepared from 2–3 male GRP::eGFP transgenic mice as described above. For staining, the sections were incubated overnight at 4 °C with a mixture of primary antibodies diluted in Tris buffer containing 2% normal goat serum. Sections were washed extensively and incubated for 1 h at room temperature with the corresponding secondary antibodies conjugated to Cy3 (1:500), Cy5 (1:200) (Jackson ImmunoResearch) or Alexa488 (1:1000, Molecular Probes, Eugene, OR). Sections were washed again and cover-slipped with fluorescence mounting medium (DAKO, Carpinteria, CA).

Double-immunofluorescence signals were visualized by confocal microscopy (LSM 710; Zeiss AG, Jena, Germany) using a 63x Plan-Apochromat objective (N.A. 1.4). The pinhole was set to 1 Airy unit for each channel and separate color channels were acquired sequentially. The acquisition settings were adjusted to cover the entire dynamic range of the photomultipliers. Typically, stacks of confocal images (1024 × 1024 pixels) spaced by 0.3 μm were acquired at a magnification of 56–130 nm/pixel. For display, images were processed with the image analysis software Imaris (Bitplane; Zurich, Switzerland). Images from all channels were overlaid (maximal intensity projection) and background was subtracted, when necessary. A low-pass filter was used for images displaying α subunit staining. Analysis of the distribution of α subunit-IR in GRP::eGFP neurons and dendrites and MrgprA3::cre-eGFP axons was performed in single confocal sections acquired at a magnification of 78 nm / pixel in 8-bit gray scale images, using a threshold segmentation algorithm (minimal intensity, 90–130; area > 0.08 μm^2^). Distribution of GABA_A_Rα3 protein was studied in brain and spinal cord sections obtained from adult hoxB8-α3^resc/resc^, global α3^−/−^, and wild-type mice. For immunoperoxidase staining, a polyclonal antibody directed against the N-terminal fifteen amino acids (pGluGESRRQEPGDFVKQ) of the rat GABA_A_R α3 protein was used as the primary antibody. Sections from GABA_A_R-mutated mice and from controls were treated in a strictly parallel fashion.

### RNAscope fluorescent in situ hybridization

Multiplex fluorescent in situ hybridization was performed using RNAscope (Advanced Cell Diagnostics; ACD; ref. ^59^). In brief, dissected tissue was snap frozen in liquid nitrogen and later on embedded in NEC50. Thirty five µm sections were cut on a Hyrax C60 cryostat, mounted on superfrost^+^ glass slides and stored at −80 °C until use. The in situ hybridization was carried out according to the manufactures protocol. The following RNAscope probes were used: GABA_A_R α1 (catalog number: 435351); GABA_A_R α2 (435011-C2); GABA_A_R α3 (435021-C3); GABA_A_R α5 (319481); GRP (317861 and 317861-C2); GRPR (317871 and 317871-C2); EGFP (400281 and 400281-C3). The latter was used to detect eGFP expressed under the genetic control of the MrgprA3 gene in MrgprA3::cre-eGFP transgenic mice.

### Human spinal cord tissue samples

Tissue has been extracted during routine autopsy from five patients. Two samples had been formalin fixed and paraffin embedded, and three were fresh frozen tissue samples. All samples were screened with control RNAscope fluorescent in situ hybridization probes provided by the manufacturer of the assay [human Polr2a (C1), PPIB (C2) and UBC (C3)]. Tissue samples of one patient were well enough preserved to yield reliable and specific in situ hybridization signals. These samples were from a 37 years old male patient with a post mortem time of 16 h who had died of septic shock and right heart failure. From this patient, small samples from the cervical, thoracic and lumbar spinal cord had been fixed in 4% formalin for 14 days, and were then paraffin-embedded. Ten micrometer thick slices were mounted on superfrost glass slides. Multiplex fluorescent in situ hybridization on human spinal cord tissue was performed using RNAscope® Multiplex Fluorescent Reagent Kit Version 2 (323100, Advanced Cell Diagnostics; ACD; ref.^59^). The in situ hybridization was carried out according to the manufactures protocol with the following modifications. Target retrieval was carried out at 94–98 °C for 30 min in target retrieval buffer. Protease incubation was carried out for 30 min at 40 °C with the protease plus reagent. The following RNAscope probes were used: α2GABA_A_R (Hs-GABRA2 448211-C2), α3GABA_A_R (Hs-GABRA3 320269-C3), GRP (Hs-GRP 465261-C1) and GRPR (Hs-GRPR 460411-C1) were used to detect co-expression of GRP / GRPR and α2GABA_A_R or α3GABA_A_R subunit transcripts.

### Skin histology and immunofluorescence

Inflamed and healthy back skin was collected. Tissues were embedded in OCT compound (Sakura Finetek, Torrance, USA) and frozen on dry ice. Cryostat sections (7 μm) were placed on glass slides, air dried, fixed with acetone for 2 min at −20 °C and subsequently rehydrated with 80% methanol for 5 min at 4 °C. Specimens were incubated with 5% donkey serum, 0.1% Triton-X and 1% BSA in PBS for 1 h at room temperature, followed by overnight incubation with rat anti-mouse CD68 (1:200; Abcam, Cambridge, United Kingdom) at 4 °C. The samples were incubated with Alexa Fluor 488- or 594-coupled secondary antibodies and Hoechst 33342 (all from Invitrogen, Life Technologies, Carlsbad, USA) for 30 min at room temperature. CD68-stained sections were examined on an Axioskop 2 mot plus microscope (Carl Zeiss, Feldbach, Switzerland), equipped with an AxioCam MRC camera (Zeiss) and a Plan-Apochromat 0.45 NA ×10 objective (Zeiss). Images of at least four individual fields of view were acquired per section using Axio-Vision software 4.8. Using ImageJ v1.49, the fluorescent area was determined between the stratum corneum and an outline thereof shifted 300 µm into the tissue. Results are expressed as CD68-positive area (µm^2^) per µm basement membrane.

### Electrophysiological recordings in HEK293 cells recordings

The effects of TPA023B on currents through recombinant GABA_A_Rs were studied in HEK293 cells (ATCC) transiently expressing GABA_A_Rs. HEK293 cells were transfected using lipofectamine LTX^28^. To ensure expression of the γ2 subunit (required for modulation of GABA_A_Rs by BDZs) in all recorded cells, we transfected cells with a plasmid expressing the γ2 subunit plus eGFP from an IRES, and only selected eGFP-positive cells for recordings. The transfection mixture contained (in µg): 1 α1, 1 β2, 3 γ2/eGFP (used as a marker of successful transfection) or 1 αx, 1 β3, 3 γ2/eGFP in case of α2, α3, or α5GABA_A_Rs. Recordings were made 18–36 h after transfection. Whole-cell patch-clamp recordings of GABA-evoked currents were made at room temperature (20–24 °C) and at a holding potential of −60 mV. Recording electrodes were filled with solution containing (in mM): 120 CsCl, 10 EGTA, 10 HEPES (pH 7.40), 4 MgCl_2_, 0.5 GTP and 2 ATP. The external solution contained (in mM): 150 NaCl, 10 KCl, 2.0 CaCl_2_, 1.0 MgCl_2_, 10 HEPES (pH 7.4), and 10 glucose. GABA was applied to the recorded cell using a manually controlled pulse (4–6 s) of a low sub-saturating GABA concentration (EC_5_). EC_5_ values of GABA were determined for all subunit combinations analyzed. EC_50_ values and Hill coefficients (n_H_) were obtained from fits of normalized concentration-response curves to the equation I_GABA_ = I_max_ [GABA]^nH^/([GABA]^nH^ + [EC_50_]^nH^). I_max_ was determined as the average maximal current elicited by a concentration of 1 mM GABA. TPA023B was dissolved in DMSO and subsequently diluted with recording solution was co-applied together with GABA without preincubation.

### Primary pruritoceptive neuron preparation and recordings

Lumbar dorsal root ganglia (DRGs) were dissected from 6–8 weeks old MrgprA3::cre-eGFP mice as previously described^60^. After removal of the connective tissue, DRGs were incubated twice in Liberase^TM^ DL Research Grade (0.09 mg ml^−1^, Roche) for 30 min. DRGs were washed with phosphate buffered saline PBS (Gibco) and incubated with Trypsin-EDTA (Gibco) for 15 min. After washing with TNB^TM^ medium (Biochrom) supplemented with L-glutamine (Gibco), Protein-Lipid-Komplex (Biochrom), penicillin-streptomycin (PenStrep, Gibco), DRGs were mechanically dissociated with fire-polished Pasteur pipettes and centrifuged through a 3.5 % BSA cushion (Sigma) to remove non-neuronal cells. The cell pellets were resuspended in supplement TNB^TM^ medium and plated on coverslips coated with poly-L-lysine and laminin (Sigma). The sensory neurons were cultured in supplement TNB^TM^ medium containing mNGF 2.5 S (Alomone Labs, 10 µg / 100 ml TNB medium) at 37 °C in 5% CO_2_. Within 48–72 h after plating, whole-cell path-clamp recordings were performed. TNB^TM^ medium was replaced with extracellular solution containing (in mM): 150 NaCl, 10 KCl, 2.0 CaCl_2_, 1.0 MgCl_2_, 10 HEPES (pH 7.4), and 10 glucose. GABA_A_R membrane currents were recorded from eGFP positive neurons at a membrane potential of −70 mV. Patch pipettes (borosilicate glass; 3–4 MΩ) were filled with intracellular solution containing (in mM): 120 CsCl, 2 MgCl_2_, 6 H_2_O, 10 HEPES, 0.05 EGTA, 2 MgATP, 0.1 NaGTP (pH 7.35). GABA_A_R currents were evoked by application of 5–20 µM GABA. TPA023B was dissolved in DMSO and applied at the concentration of 1 µM.

### Electrophysiological recordings in spinal cord slices

Transverse spinal cord slices (400 µM thick) were prepared from 20 to 29-day old GRP::eGFP mice of either sex. Slices were cut in an ice-cold solution containing (in mM): 130 K-gluconate, 15 KCl, 0.05 EGTA, 20 HEPES, and 25 glucose, pH 7.4 (adjusted with NaOH). D-2-amino-5-phosphonovaleric acid, 50 µM) was added to prevent glutamate toxicity. Slices were maintained in artificial CSF (32 °C) containing (in mM): 125 NaCl, 2.5 KCl, 1.25 NaH_2_PO_4_, 26 NaHCO_3_, 25 glucose, 2 CaCl_2_, and 1 MgCl_2_ (equilibrated with 95% O_2_, 5%CO_2_)^61^. Whole-cell patch clamp recordings were made at room temperature targeting eGFP positive neurons. During recordings, slices were continuously superfused at the rate of 1–2 ml min^−1^ with aCSF containing (in mM): 120 NaCl, 2.5 KCl, 1.25 NaH_2_PO_4_, 26 NaH_2_CO_3_, 5 HEPES, 1 MgCl_2_, 2 CaCl_2_ and 14.6 glucose (pH 7.4), equilibrated with 95% O2, 5% CO2. Recorded neurons were voltage clamped at −70 mV using an EPC 9 amplifier (HEKA Elektronic, Lambrecht, Germany) controlled with Patchmaster acquisition software. Patch pipettes (borosilicate glass; 3.5–4.5 MΩ) were filled with intracellular solution containing (in mM): 120 CsCl, 2 MgCl_2_, 6 H_2_O, 10 HEPES, 0.05 EGTA, 2 MgATP, 0.1 NaGTP, 5 QX-314 (pH 7.35). IPSCs were evoked by electrical stimulation (300 µs, 0.2–50 V) at 0.05 Hz using glass electrode filled with aCSF and placed 50–100 µm from the soma of the recorded cell. Experiments were performed in the presence of NBQX (20 µM), AP5 (50 µM), and strychnine (0.5 µM), in order to isolate the GABAergic component of IPSCs. At the end of the recordings bicuculline (10 µM) was added to confirm the GABAergic nature of the recorded IPCSs. The weighted decay time constant (τ_w_) was calculated from dual-exponential fits using the following equation: τ_w_ = (τ_1_A_1_ + τ_2_A_2_)/(A_1_ + A_2_) where τ_1_ and τ_2_ are the fast and the slow decay time constants and A_1_ and A_2_ are the equivalent amplitude weighting factors^62^. Access resistance of each neuron was continuously monitored with short hyperpolarizing voltage step applied before the electrical stimulation. Cells in which the access resistance changed more than 20 % were excluded from the analysis.

### [^3^H]Ro 15-4513 binding

Brain tissue from 8–10 week old quadruple RRRR (H → R) point mutated mice, in which all high-affinity diazepam-sensitive binding were inactivated, was homogenized in 20 volumes of 10 mM Tris-HCl, pH 7.4, 100 mM KCl containing protease inhibitors (Complete Mini, Roche Diagnostics) and centrifuged at 1000 g for 10 min. The supernatant was centrifuged for 20 min at 30,000 × *g* and the resulting crude membrane pellet was washed once with buffer. For radioligand binding, aliquots of the crude membranes (100 µg) were incubated with increasing concentrations of diazepam (binds to diazepam-sensitive sites), bretazenil (binds to diazepam-sensitive and insensitive sites^63^) or TPA023B and 4 nM [^3^H]Ro 15-4513 (22.7 Ci mmol^−1^, PerkinElmer, binds to diazepam-sensitive and insensitive sites) in a total volume of 0.2 ml for 90 min on ice. Non-specific [^3^H]Ro 15–4513 binding was assessed by addition of 10 µM flumazenil to the reaction. Incubation was stopped by rapid vacuum filtration using a semiautomatic cell harvester (Skatron Instruments) and washed filters were subjected to liquid scintillation counting.

### Behavioral experiments in mice

All behavioral experiments were performed in 7–12-week-old female and male mice. Care was taken to ensure equal numbers of female and male mice. All behavioral experiments were made by an experimenter, blinded either to the genotype of the mice or to their treatment with drug or vehicle. In experiments involving comparisons between diazepam or TPA023B and vehicle, mice were randomly assigned to the different groups. No formal sample size calculation was made. Group sizes were chosen based on previous experience with the respective behavioral test. Mechanical sensitivity was assessed with electronic von Frey filaments (no. 7; IITC, Woodland Hills, CA) and quantified as the change in the paw withdrawal thresholds measured in g. Heat hyperalgesia was evaluated in the Hargreaves test as the change in the latency of paw withdrawal to a defined heat stimulus. Responses to light mechanical stimulation of the hairy skin was tested as the change in the paw withdrawal responses upon gentle stimulation with a paint brush using the following score: 0 (no evoked movement), 1 (walking away or brief paw lifting of 1 s or less), 2 (sustained lifting of more than 2 s), 3 (strong lateral lifting above a 90° angle) or 4 (flinching/ licking of the affected paw). Six measurements were made for each animal for all three tests.

### Acute itch

was assessed in mice that received intradermal microinjections of pruritogens or 0.9% saline into the right cheek, which had been shaved at least 1 day before the experiment. In two sets of experiments that addressed the contribution of GABA_A_Rs on primary and secondary pruritoceptors, pruritogens were injected into the skin of the left thigh (Fig. 7a–c). Before injection, mice were acclimatized to a 15 cm diameter cylindrical enclosure for more than 30 min with cage bedding on the floor. Background white noise generated by a radio at ambient volume was applied to prevent auditory distraction. A 30 gauge needle was inserted bevel-up and pushed 5 mm horizontally into the skin beyond the point of insertion, before injection of the pruritogen (in a total volume of 10 µl). No anesthesia was used. Correct injection was confirmed by the appearance of a slightly domed bulla. After injection, mice were placed back into the cylindrical enclosure and videotaped for 30 min. Videos were analyzed off-line. Scratching with the hind paw directed to the ipsilateral cheek was counted in bouts, with one bout defined as an instance when the mouse lifted its paw to scratch until it returned the paw to the cage floor. In case of experiments in which the pruritogen was injected into the skin of the thigh, the time spent biting the injected skin area was counted in s min^−1^ as a measure of itch.

### Chronic itch

was investigated in the contact dermatitis model^27^ and the dry skin model^30^. To induce contact dermatitis, mice were treated on day 0 with 10% oxazolone in acetone/olive oil (4:1 v v^−1^) on the shaved nape of the neck (100 µl). After a resting period of 7 days, mice were treated with 1% oxazolone in acetone/olive oil (4:1 v v^−1^) on the nape of the neck (100 µl) every other day for 10 days. On the day of the experiment, mice were injected with drug or vehicle i.p. under short light isoflurane anesthesia. Scratching of the hind paw directed to the ipsilateral cheek was quantified as the number of scratching bouts. In the dry skin model, mice were treated with a mixture of acetone and diethylether (1:1) on the shaved nape of the neck for 15 s, followed by distilled water for 30 s, twice daily for 10 days. On the day of the experiment, vehicle or drug was administered i.p. under short light isoflurane anesthesia.

To quantify the severity of skin lesions a dermatitis score was determined^64^. Hemorrhage/erythema, dryness/scaring, and hyperplasia were scored as 0 (none), 1 (mild), 2 (moderate), or 3 (severe) once per day resulting in a score between 0 and 9. Photographs of atopic dermatitis-like skin were taken before treatment (day 11) and 9 days after treatment (day 20).

### Locomotor activity, motor coordination and muscle relaxation

TPA023B (1 mg kg^−1^, p.o. or i.p.) or vehicle was administered 60 min before the tests. Locomotor activity was measured in an open field arena (10 cm radius) equipped with four pairs of light beams and photosensors and analyzed for the time interval between 60 and 120 min after TPA023B administration. Motor coordination was assessed with a rotarod accelerating from 4 r.p.m. to 40 r.p.m. within 5 min. Fifteen measurements were taken per mouse. To assess muscle relaxation, mice were placed with their forepaws onto a metal horizontal wire placed 20 cm above ground. Successes and failures to grab the wire with at least one hindpaw were recorded between 60 and 120 min after TPA023B administration.

### Pruritus study in dogs

Twelve 6 months-old beagle dogs (6 females, 6 males) weighing between 9.0 and 13.0 kg (see Supplementary Table 1) were included in the study. They were sensitized using lyophilized extracts of the house dust mite *Dermatophagoides farinae* in mineral oil. In order to expose the living epidermis, the skin of the abdomen was tape-stripped. *D. farinae* extract was gently applied on the tape-stripped skin once a week for eight weeks. At this time point, dogs were considered *D. farinae*-sensitized even though most of them (8 out of 12) did not exhibit pruritus or clinical signs of atopic dermatitis. Four weeks later, the dogs were challenged using *D. farinae* extracts after tape stripping of the abdominal skin. This challenge was made on three consecutive days (days 1 to 3). Pruritus and clinical signs were assessed on day 2, 3, and 4. All dogs exhibited pruritus and clinical signs compatible with atopic dermatitis on the site of challenge but also in remote areas. After the third challenge all dogs exhibited histological signs of atopic dermatitis and nine dogs showed positive reactions in intradermal allergen exposure test. These nine dogs were enrolled to assess the antipruritic effect of TPA023B in a randomized placebo-controlled observer-blind cross-over study. TPA023B was administered at a dose of 20 mg per dog as a tablet, 30 min after the challenge. For the following 6 h, dogs were kept in groups of three in closed arenas of 2 × 4 m and continuously video recorded. Both the number of scratching bouts and the total time spent scratching per hour were determined. Recordings were made on the day of the second challenge (baseline) and on the following day after drug or placebo administration. The same procedure was done three weeks later with switched treatment (TPA023B and placebo). Number of scratching bout and total time spent scratching were normalized to the control values obtained the day before (second challenge). We did not correct our outcome parameters for baseline scratching present in the absence of the pruritogenic challenge. Blood samples were taken from all dogs at the end of the video recording and whole blood concentrations of TPA023B were measured by high performance liquid chromatography/high resolution electrospray time-of-flight mass spectrometry to verify drug exposure.

### Statistics

For most experiments, results of individual mice or cells are displayed as individual symbols. Normal distribution of data was assumed when t-tests or ANOVAs were applied.

### Data availability

Excel files including the data that support the findings of this study are available at G-Node.org with the identifier doi: 10.12751/g-node.fb5bd5 [https://doid.gin.g-node.org/fb5bd596a1ff2f6e8cd29f51be351ad3/]. Additional raw data of this study are from the corresponding author upon reasonable request.

## Electronic supplementary material

Supplementary Information

Description of Additional Supplementary Files

Supplementary Movie 1

Supplementary Movie 2

Supplementary Movie 3

Supplementary Movie 4
