# Supplementary material for: Itch suppression in mice and dogs by modulation of spinal α2 and α3GABAA receptors
Source: Nat Commun. 2018 Aug 13;9:3230. doi: 10.1038/s41467-018-05709-0 (PMC6089996; doi:10.1038/s41467-018-05709-0)
Supplement: Supplementary file 1 — Supplementary Information [file 41467_2018_5709_MOESM1_ESM.pdf]

Supplementary Information

Itch suppression in mice and dogs by selective modulation of spinal $\alpha 2$ and $\alpha 3$ $GABA_A$ receptors

Ralvenius et al.

Supplementary Figures

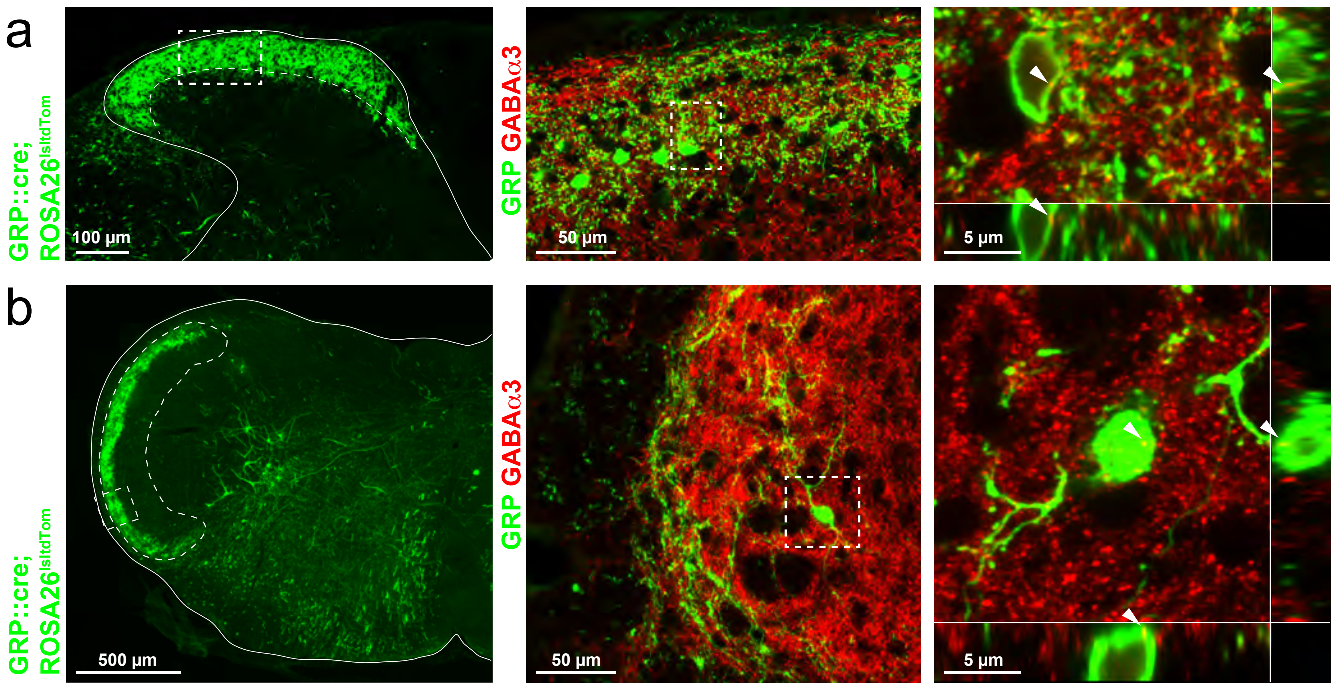

Supplementary Figure 1. α3GABA$_A$R expression in the mouse cervical spinal cord and medullary dorsal horn.
Left: low magnification overviews illustrate the localization of GRP neurons in both structures. Dashed lines indicate the border between cervical lamina II and III (in a) and of the spinal trigeminal nucleus (in b). Dashed squares indicate areas displayed at high magnification in the middle panels. Middle: GRP positive cells (green) overlap with α3GABA$_A$R immunoreactivity (red). Right: high magnification and orthogonal views indicate localization α3GABA$_A$Rs on GRP expressing cells (see arrow heads). Similar results were obtained in three mice.

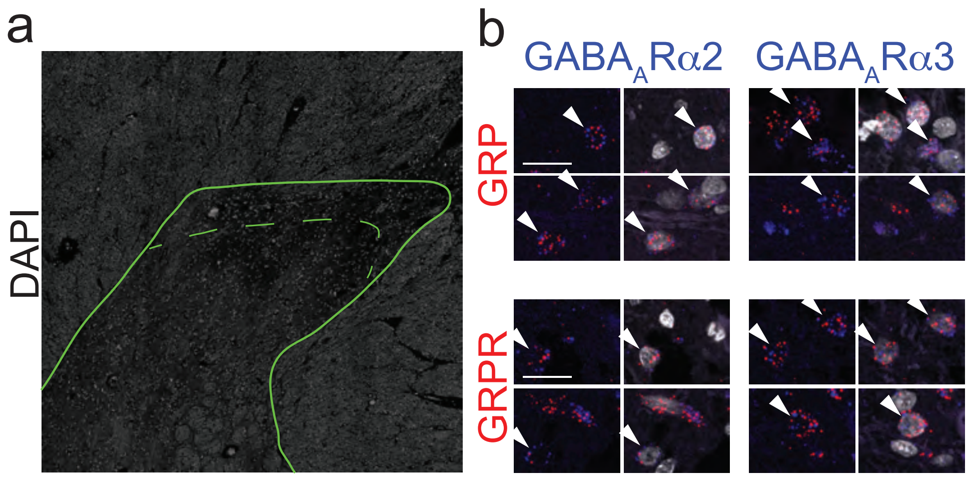

Supplementary Figure 2. GABA$_A$R α2 and α3 subunit transcripts in GRP and GRPR neurons of the human spinal cord.
(**a**) Transverse section of the human cervical dorsal horn (grey matter border indicated in green). GRP and GRPR expressing neurons were found in the superficial dorsal horn. (**b**) Co-expression of GRP or GRPR (red dots) with α2 or α3GABA$_A$R subunits (blue). Arrows indicate cells co-expressing GRP or GRPR with α2 or α3GABA$_A$R subunits. DAPI positive nuclei are illustrated in white. Scale bars: 20 µm.

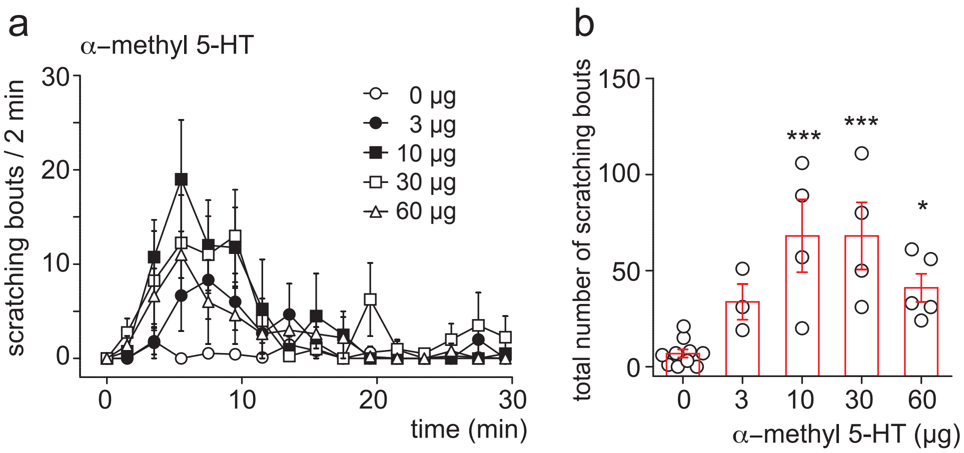

Supplementary Figure 3. Dose finding for α-methyl 5-HT in 129 SvJ mice.
(**a**) Scratching responses in wild-type 129X1/SvJ evoked by the metabolically more stable serotonin analog α-methyl 5-HT. Different doses of α-methyl 5-HT were injected intracutaneously into the right cheek and scratching responses were counted for 30 min. Number of scratching bouts (mean ± SEM) over time following injection of α-methyl 5-HT. (**b**) Quantification and statistical analyses (ANOVA followed by Dunnett's post hoc test). Circles are values obtained from individual mice. Bars and error bars (red) are means and SEM. $F(4,21) = 8.84$; *, $P < 0.05$; ***, $P < 0.001$; n = 10, 3, 4, 4, and 5, for 0, 3, 10, 30, and 60 µg, respectively.

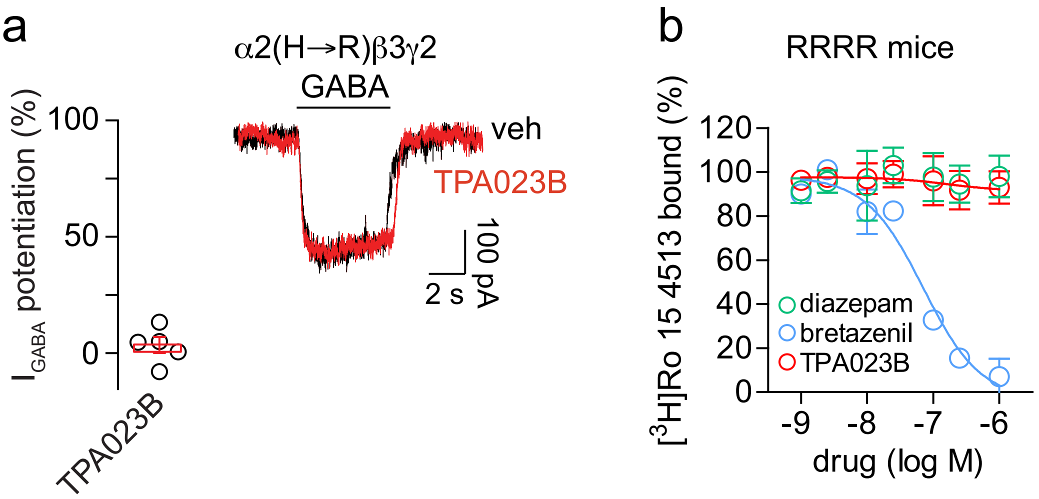

Supplementary Figure 4. The H → R point mutation in GABA$_A$R α subunits prevents modulation by and binding of TPA023B.
(**a**) Positive allosteric modulation by TPA023B (1 µM) of α2GABA$_A$Rs in HEK 293 cells is abolished by the H → R point mutation in the α2 subunit (n = 5). For data in wild-type GABA$_A$Rs see figure 4a. (**b**) Accordingly, TPA023B failed to displace [^{3}H]Ro 15-4513 binding to brain membranes prepared from GABA$_A$R quadruple point mutated (RRRR) mice. The same was confirmed for diazepam, which only binds to wild-type receptors. In contrast, bretazenil, which binds to both wild-type and α(H→R) point mutate receptors, dose-dependently inhibited [^{3}H]Ro 15-4513 binding. n (number of cells) = 4. Error bars indicate s.d. Error bars smaller than the symbol are not visible.

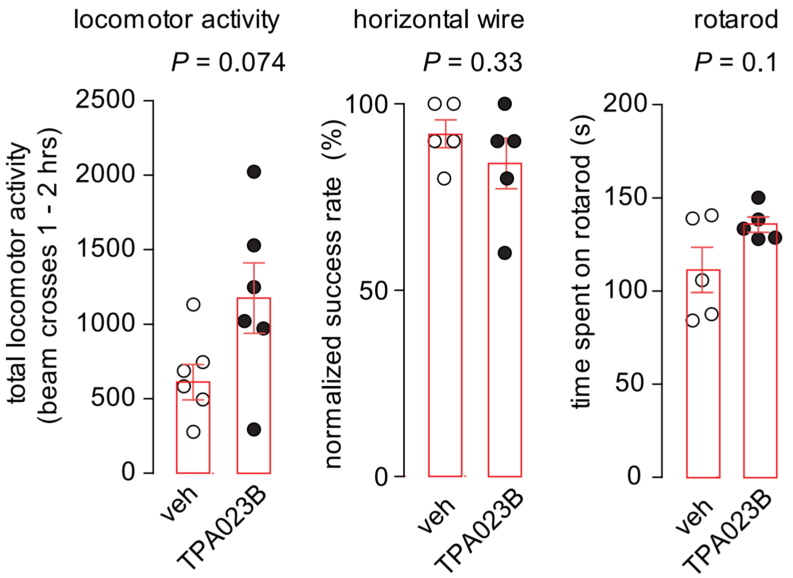

Supplementary Figure 5. Behavioral effects of TPA023B (1 mg/kg, i.p.) in wild-type mice.
Locomotor activity in the open field test. Statistical analysis of total number of beam crosses within the time interval 60 - 120 min (*P* = 0.074, unpaired t-test, n = 6). Muscle relaxation in the horizontal wire test (*P* = 0.33, unpaired t-test, n = 5). Motor coordination in the rotarod test (average time spent on the rotarod (*P* = 0.1, paired t-test, n = 5). Error bars indicate s.e.m.

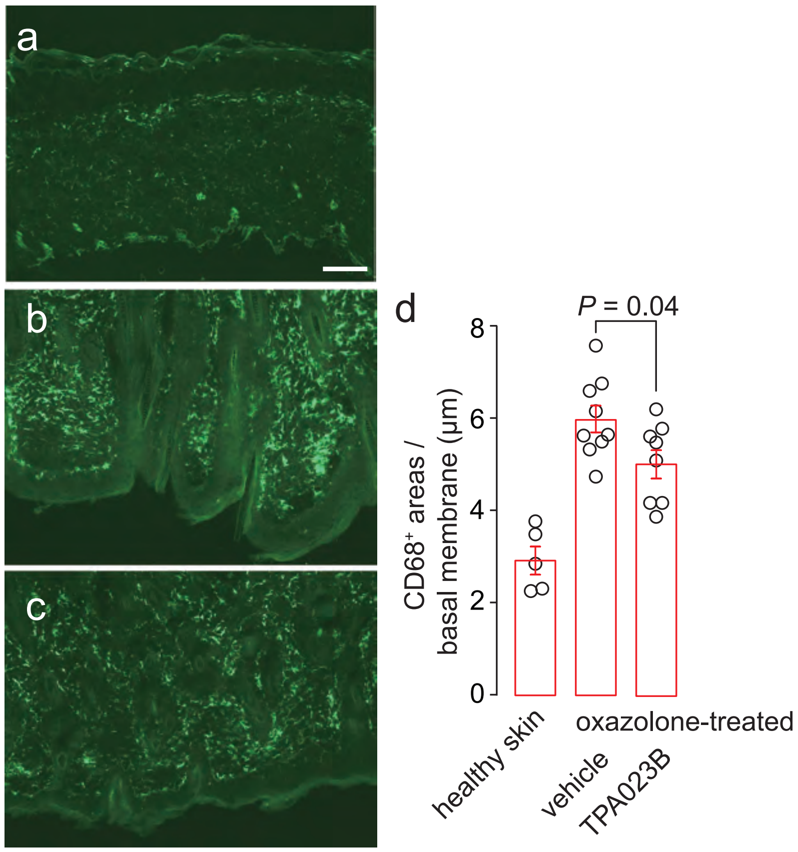

Supplementary figure 6. Chronic treatment with TPA023B reduces macrophage infiltration.
(**a**) Skin samples from healthy mice (**a**), and atopic-like (oxazolone-treated) mice treated for nine days either with vehicle (**b**) or TPA023B (1 mg/kg, i.p.) (**c**). Skin samples were taken from the mice used for the experiments shown in figure 5. Infiltrating macrophages were visualized with an antiserum against CD68. Scale bar, 100 µm. (**d**) Quantification. Statistical comparison was performed between vehicle and TPA023B treated atopic mice. Macrophage infiltration in healthy mice (left column, n = 5) is shown for comparison. *P* = 0.04, unpaired t-test, n = 9 and 8, for vehicle and TPA023B, respectively.

Supplementary Tables

Gene symbol	Mouse Marker Name	Chromo-some	Fraction marker positive cells sensory neuron population NP1 =MrgprD	NP2 =MrgprA3	NP3 =BNP
Gabra1	$GABA_A$ receptor, subunit alpha 1	11	0.224	0.0625	0
Gabra2	$GABA_A$ receptor, subunit alpha 2	5	0.08	0.0938	0
Gabra3	$GABA_A$ receptor, subunit alpha 3	X	0	0.0313	0
Gabra4	$GABA_A$ receptor, subunit alpha 4	5	0	0	0
Gabra5	$GABA_A$ receptor, subunit alpha 5	7	0.016	0	0
Gabra6	$GABA_A$ receptor, subunit alpha 6	11	0	0	0

Supplementary Table 1. $GABA_AR$ α subunit expression in three populations of primary pruritoceptive neurons. Data extracted from Supplementary Reference [1]. The NP1, NP2 and NP3 populations are largely identical with the MrgpRD, MrgprA3 and BNP positive subpopulations, respectively.

Scratching bouts (number/hour)

	dog 1	dog 2	dog 3	dog 4	dog 5	dog 6	dog 7	dog 8	dog 9	
Chip	"4154"	"0428"	"8959"	"5543"	"0788"	"1177"	"0483"	"2584"	"4721"	
sex	male	male	male	male	male	male	female	female	female	
weight (kg)	13.0	12.0	12.0	13.0	11.0	12.5	9.0	10.5	13.0	
round 1: day 2	8.3	1.8	1.5	5.0	4.3	6.7	6.5	7.0	7.3	
day 3	7.0	3.5	2.3	**2.8**	7.0	7.8	**2.8**	**2.2**	6.5	
round 2: day 2	5.3	2.5	3.5	8.2	7.8	6.0	3.2	5.8	11.0	
day 3	**4.2**	**1.5**	**2.5**	8.8	**3.2**	**5.2**	7.3	7.8	**5.0**	
normalized data:										
round 1: day 3/day 2	0.84	1.91	1.56	**0.57**	1.62	1.18	**0.44**	**0.31**	0.89	
round 2: day 3/day 2	**0.78**	**0.60**	**0.71**	1.08	**0.40**	**0.86**	2.32	1.34	**0.45**	
										mean ± sem
placebo	0.84	1.91	1.56	1.08	1.62	1.18	2.32	1.34	0.89	1.41 ± 0.16
TPA023B	0.78	0.60	0.71	0.57	0.40	0.86	0.44	0.31	0.45	0.57 ± 0.06
% reduction	7.0	68.6	54.1	47.6	75.0	26.7	81.2	77.0	48.7	54 ± 8.3

Time spent scratching (min/hour)

	dog 1	dog 2	dog 3	dog 4	dog 5	dog 6	dog 7	dog 8	dog 9	
round 1: day 2	89.0	9.8	4.8	30.2	30.3	69.5	22.5	19.0	36.2	
day 3	76.5	24.5	9.7	**21.8**	93.2	84.8	**4.7**	**0.2**	27.8	
round 2: day 2	70.7	13.5	21.2	94.3	155.5	79.8	19.0	38.0	137.7	
day 3	**32.7**	**9.7**	**23.5**	86.5	**50.2**	**65.3**	35.8	15.8	**62.2**	
normalized data:										
round 1: day 3/day 2	0.86	2.49	2.00	**0.72**	3.07	1.22	**0.21**	**0.01**	0.77	
round 2: day 3/day 2	**0.46**	**0.72**	**1.11**	0.92	**0.32**	**0.82**	1.89	0.42	**0.45**	
										mean ± sem
placebo	0.86	2.49	2.00	0.92	3.07	1.22	1.89	0.42	0.77	1.51 ± 0.30
TPA023B	0.46	0.72	1.11	0.72	0.32	0.82	0.21	0.01	0.45	0.54 ± 0.11
% reduction	46.2	71.3	44.5	21.1	89.5	33.0	89.0	97.9	41.3	59 ± 9.4

Supplementary Table 2. Antipruritic responses to TPA023B in individual dogs.
Bold numbers are values obtained following treatment with TPA023B (20 mg, p.o.)

Mouse line	Forward primer	Reverse primer
α1(H→R)<br>Gabra1$^{tm1.1Uru}$	UR12: CAA TGG TAG GCT CAC TCT GGG AGA TGA TA	UR 70: AAC ACA CAC TGG CAG GAC TGG CTA GG
α2(H→R)<br>Gabra2$^{tm1.1Uru}$	KL25: GCA TGC ACC ACC CAG GAA GCG ATT	KL5: 5TCC ATC ATC CTG GAT TCG AAG CAG C
α3(H→R)<br>Gabra3^{tm1Uru}	UR75: GAC AGA CAT GGC ATG ATG AAA GAC TGA AAT	UR106: ACA AAA TGT AAG AAC AAG AAC CAA GAA AAT
α5(H→R)<br>Gabra3^{tm2Uru}	RK4: TTA AAC CGC AGC CTT TCA TCT TTC	RK5: GAG GCC ACC TAA TGC TTC CAG CTT
α2^{floxed}<br>Gabra2$^{tm2.1Uru}$	R24: GTC CTA TCC CTT CGT CCT CAC C	R23: TAT CTT GTC TTT CCC CTC CTG GTT G
α3$^{-/-}$<br>Gabra3^{tm2Uru}	UR75: GAC AGA CAT GGC ATG ATG AAA GAC TGA AAT	UR106: ACA AAA TGT AAG AAC AAG AAC CAA GAA AAT
sns-cre<br>Tg(Scn10a-cre)1Rkun	Cre A: GCA TGA TCT CCG GTA TTG AAA CTC C	CreS: TGA CAG CAA TGC TGT TTC ACT GG
hoxB8-cre<br>Tg(Hoxb8-cre)1403Uze	o649 HoxB8_s: GCC TCA AAA TTC AAT AAA ACG CCA CC	o235 CreA: GCA TGA TCT CCG GTA TTG AAA CTC C
mrgprA3::cre<br>Tg(Mrgpra3-GFP/cre)#Xzd	BACA3-F789: TAT CAT GGC CGA CAA GCA GAA	BACA3-R1287: CCG GTT ATT CAA CTT GCA CCA T
GRP::eGFP<br>Tg(Grp-EGFP)DV197Gsat/Mmucd	Grp (10444) F1: GGG ACA ACG CAC TCT CAG CCT A	GFP R2: TAG CGG CTG AAG CAC TGC A
GRPR::eGFP<br>Tg(Grpr-EGFP)PZ62Gsat/Mmucd	Grpr (36178) F1: GAT CAG CGA GCC TAA CTG ACA AAC C	GFP R3: GGT CGG GGT AGC GGC TGA A
GRP::cre<br>Tg(Grp-cre)KH288Gsat/Mmucd	Grp F: GGG ACA ACG CAC TCT CAG CCT A	CreGS R1: Cgg CAA ACg gAC AgA AgC ATT
ROSA26$^{lsl-TVA}$<br>Gt(ROSA)26Sor<tm1(Tva)Dsa	R26-Tva-UP: AAAGTCGCTCTGAGTTGTTAT	R26-Tva-SA-mut-LP: GCGAAGAGTTTGTCCTCAACC<br>R26-Tva-WT-LP: GGAGCGGGAGAAATGGATATG
ROSA26$^{lsl-tdTom}$ Gt(ROSA)26Sor<br>tm66.1(CAG-tdTomato)Hze/J	oIMR9105: CTG TTC CTG TAC GGC ATG G	oIMR9103: GGC ATT AAA GCA GCG TAT CC

Supplementary Table 3. Mutant mouse strains and PCR primers for genotyping.

Supplementary References:

1. Usoskin, D., *et al.* Unbiased classification of sensory neuron types by large-scale single-cell RNA sequencing. *Nat Neurosci* **18**, 145-153 (2015).
