# Supplementary material for: Itch suppression in mice and dogs by modulation of spinal α2 and α3GABAA receptors
Source: Nat Commun. 2018 Aug 13;9:3230. doi: 10.1038/s41467-018-05709-0 (PMC6089996; doi:10.1038/s41467-018-05709-0)
Supplement: Supplementary file 2 — Description of Additional Supplementary Files [file 41467_2018_5709_MOESM2_ESM.pdf]

**Description of Additional Supplementary Files**

File Name: Supplementary Movie 1
Description: Home cage behaviour of a RHRR ("α2 only") mouse 30 min after i.p. injection of 250 µl vehicle.

File Name: Supplementary Movie 2
Description: Home cage behaviour of a RHRR ("α2 only") mouse 30 min diazepam (10 mg/kg, i.p.).

File Name: Supplementary Movie 3
Description: Home cage behaviour of a RRHR ("α3 only") mouse 30 min after i.p. injection of 250 µl vehicle.

File Name: Supplementary Movie 4
Description: Home cage behaviour of a RRHR ("α3 only") mouse 30 min after diazepam (10 mg/kg, i.p.).
